# Supplementary material for: Randomised controlled feasibility trial of the Active Communication Education programme plus hearing aid provision versus hearing aid provision alone (ACE to HEAR): a study protocol
Source: BMJ Open. 2018 Aug 1;8(7):e021502. doi: 10.1136/bmjopen-2018-021502 (PMC6074637; doi:10.1136/bmjopen-2018-021502)
Supplement: Author's manuscript [file bmjopen-2018-021502.draft_revisions.pdf]

BMJ Open is committed to open peer review. As part of this commitment we make the peer review history of every article we publish publicly available.

When an article is published we post the peer reviewers' comments and the authors' responses online. We also post the versions of the paper that were used during peer review. These are the versions that the peer review comments apply to.

The versions of the paper that follow are the versions that were submitted during the peer review process. They are not the versions of record or the final published versions. They should not be cited or distributed as the published version of this manuscript.

BMJ Open is an open access journal and the full, final, typeset and author-corrected version of record of the manuscript is available on our site with no access controls, subscription charges or pay-per-view fees (http://bmjopen.bmj.com).

If you have any questions on BMJ Open's open peer review process please email info.bmjopen@bmj.com

BMJ Open

A randomised controlled feasibility trial of the Active Communication Education programme plus hearing-aid provision versus hearing aid provision alone (ACE To HEAR): A study protocol.

Journal:	*BMJ Open*
Manuscript ID	bmjopen-2018-021502
Article Type:	Protocol
Date Submitted by the Author:	09-Jan-2018

Complete List of Authors:	Thyer, Nicholas; University of Leeds, Leeds Institute of Cardiovascular and Metabolic Medicine (LICAMM)<br>Watson, Jude; University of York, Department of Health Sciences<br>Jackson, Cath; Valid Research<br>Hickson, Louise; The University of Queensland, Department of Speech and Hearing<br>Maynard, Christina; University of Leeds, Leeds Institute of Cardiovascular and Metabolic Medicine (LICAMM)<br>Forster, Anne; University of Leeds, Academic Unit of Elderly Care and Rehabilitat<br>CLARK, LAURA; University of York, Department of Health Sciences<br>Bell, Kerry; University of York, Health Sciences<br>Fairhurst, Caroline; University of York, Health Sciences<br>Cocks, Kim; University of York, Department of Health Sciences<br>Gardner, Rob; Bradford Teaching Hospitals NHS Foundation Trust<br>Iley, Kate; York Teaching Hospital NHS Foundation Trust, Audiology<br>Gailey, Lorraine; Hearing Link

Keywords:	Hearing Loss, Active Communication Education, Hearing Aid Benefit, Economic Benefit, Communication Benefit, Hearing Aid Use

SCHOLARONE™ Manuscripts

A RANDOMISED CONTROLLED FEASIBILITY TRIAL OF THE ACTIVE COMMUNICATION EDUCATION PROGRAMME PLUS HEARING AID PROVISION VERSUS HEARING AID PROVISION ALONE (ACE TO HEAR) – A STUDY PROTOCOL.

N J Thyer,[1] J Watson,[2] C Jackson,[3] L Hickson,[4] C Maynard, [1] A Forster, [5] L Clark,[2] K Bell,[2] C Fairhurst,[2] K Cocks,[2] R Gardner[6], K Iley, [7] L Gailey. [8]

Acknowledgments: The Authors would like to thank Catherine Hewitt[2] for helpful statistical advice and comments on an earlier draft of this paper.

Corresponding author:

Dr N J Thyer: n.j.thyer@leeds.ac.uk

Author affiliations:

[1]Leeds Institute of Cardiovascular and Metabolic Medicine (LICAMM,) University of Leeds, Leeds, UK

[2]York Trials Unit, Department of Health Sciences, University of York, ARRC Building, York, UK

[3]Valid Research Ltd, Wetherby, UK

[4]School of Health and Rehabilitation Sciences, The University of Queensland, Brisbane, Queensland 4072, Australia

[5]Leeds Institute of Health Sciences (LIHS), University of Leeds, Leeds, UK

[6]Audiology Department, Bradford Royal Infirmary, Bradford, UK

[7]Audiology Department, York Hospital, York, UK

[8]Hearing Link, Eastbourne, UK

For peer review only - http://bmjopen.bmj.com/site/about/guidelines.xhtml

Abstract:

Introduction: Up-to 30% of hearing aids fitted to new adult clients are reported to be of low benefit and used intermittently or not at all. Evidence suggests that additional interventions paired with service-delivery redesign may help improve hearing aid use and benefit. The range of interventions available is limited. In particular, the efficacy of interventions like the Active Communication Education (ACE) programme that focus on improving communication success with hearing impaired people and significant others, has not previously been assessed. We propose that improved communication outcomes associated with the ACE intervention, lead to an increased perception of hearing aid value and more realistic expectations associated with hearing aid use and ownership, which are reported to be key barriers and facilitators for successful hearing aid use. This study will assess the feasibility of delivering ACE and undertaking a definitive randomised controlled trial (RCT) to evaluate whether ACE would be a cost effective and acceptable way of increasing quality-of-life through improving communication and hearing aid use in a public health service such as the NHS.

Methods and analysis: This will be a pragmatic, randomised controlled, open feasibility trial with embedded economic and process evaluations delivered in audiology departments in two UK cities. We aim to recruit 84 patients (and up to 84 significant others) aged 18 years and over, who report moderate or less than moderate benefit from their new hearing aid. The feasibility of a larger scale study and the acceptability of the ACE intervention will be measured by recruitment rates, treatment retention, follow-up rates and qualitative interviews.

Ethics and dissemination: Ethical approval granted by South East Coast-Surrey Research Ethics Committee (16/LO/2012). Dissemination of results will be via peer reviewed research publications both online and in print, conference presentations, posters, patient forums and Trust bulletins.

Trial registration number: ISRCTN28090877

For peer review only - http://bmjopen.bmj.com/site/about/guidelines.xhtml

Strengths and limitations of this study:

- This will be the first study to evaluate the processes involved in delivering the ACE intervention in a GP referral pathway for new NHS hearing aid users.
- If the RCT is shown to be feasible then the data from this study will provide critical information that will inform the design of a larger RCT to determine the social, clinical and economic outcomes of the ACE in this important clinical pathway.
- The study is powered to allow the standard deviation of the proposed outcome measures to be estimated with reasonable certainty to inform future sample size calculations.
- Ultimately the test of whether the ACE intervention leads to longer term communication success, better hearing aid use, hearing aid benefit, quality-of-life and economic gains, will be tested in a future full-scale RCT.

For peer review only

For peer review only - http://bmjopen.bmj.com/site/about/guidelines.xhtml

INTRODUCTION

Age-related hearing impairment is a major world-wide public health issue for ageing populations [1]. It is reported as the third most common chronic condition affecting approximately 328 million (91%) middle and older aged adults[2], over 10 million adults in the UK alone.[3 4] By the age of 70, 70% will have a mild or worse hearing-impairment, progressively worsening with age.[5] Hearing impairment is commonly associated with reduced quality-of-life and well-being[7-9] including depression and anxiety,[10] social isolation,[9] poor social interactions,[11-13] cognitive dysfunction,[14] increased risk of developing dementia and reduced emotional, behavioural and general social wellbeing.[15] In addition, disability in these domains is often experienced by normally-hearing significant others (SOs) living with hearing-impaired people.[16-18] Hearing impairment therefore represents an enormous burden on society and the economy.

In developed countries the most common treatment is to fit a hearing aid.[6] Despite strong evidence that hearing aid use is associated with reductions in hearing disability noted above [6 19-21] hearing aid use is remarkably low. [22 23] It is estimated that up to 30% of UK adult hearing aid owners do not use them regularly or at all.[4 24-26] International studies support these data.[8 27-29] Cost implications for the NHS are significant as they provide 80% of UK hearing aids,[25] fitting more than 300,000 new devices each year of which an estimated 20,000-120,000 are un-used. Even with global advances in technology, fitting protocols and outcome measurements,[30] there is little evidence that NHS hearing aid use and the expected gains in benefit and quality of life have improved over the last decade[19 25] and treatment continues to focus primarily on technology.

A recent systematic review of additional treatment found very low quality evidence that self-management and service delivery interventions may be of benefit in auditory rehabilitation.[31] However, the authors found no studies that examined the effect of these sorts of interventions on hearing aid use.

Reasons for hearing aid non-use are complex.[32] Research has identified psycho-social factors important for successful aural rehabilitation including; personal and societal attitudes to hearing-impairment[33] patient

For peer review only - http://bmjopen.bmj.com/site/about/guidelines.xhtml

involvement in decision making[34-37] and expectations of benefit and communication success in a range of communication situations.[38 39] Key barriers and facilitators to successful hearing aid use have been identified as being related to expectations of benefit and meaningful participation in everyday life.[40 41] The World Health Organisation's International Classification of Functioning, Disability and Health (ICF)[42] provides a functional description of difficulties (related to these expectations) experienced by hearing-impaired people and their hearing communication partners, for example, avoidance of difficult listening situations that lead to 'activity limitations' and 'participation restrictions'.

One intervention that is designed to reduce these limitations and restrictions is The Active Communication Education (ACE) programme (the focus of this paper). The ACE trains participants to develop solutions to specific difficult communication scenarios that commonly lead to their avoidance of or reduced participation in important activities. The effectiveness of ACE as an *alternative* intervention to a hearing aid has been evaluated and two small studies demonstrate ACE benefits in improving communication function and hearing related quality-of-life.[43 44] ACE effectiveness as an *adjunct* to hearing aid fitting has not been evaluated, although there is some weak evidence that supports its use in this context [44] i.e. longer-term improvements in using communication strategies being associated with hearing aid users.

A systematic review of group communication programme effectiveness conducted in 2005,[45] revealed just nine small and methodologically poor studies. The review reported weak evidence for short-term benefits related to reduced hearing disability; improvements in quality-of-life; hearing aid use and communication function[46] when interventions were delivered in concert with a hearing aid. The authors concluded that there was a clear need for large sufficiently powered randomized controlled studies to determine short and long term benefits of adult communication rehabilitation group interventions as an adjunct to hearing aid fitting. Such a study has yet to be completed. Evidence that post-dates this 2005 review does little to change the situation providing only weak additional evidence for moderate gains in hearing-loss related quality-of-life for communication-based group interventions.[47] There are indications that group

For peer review only - http://bmjopen.bmj.com/site/about/guidelines.xhtml

rehabilitation programmes like ACE have the potential to realise economic gains for service providers. For example, Abrams[48] estimated that a hearing aid plus a four-week group rehabilitation programme reduced the treatment cost per quality-adjusted life-year (QALY) gained by more than half; the cost of implementing the rehabilitation programme was less than 6% of the total rehabilitation cost per patient. Even so, with no strong evidence that such interventions delivered as an adjunct to hearing aid fitting are clinically worthwhile, they are not routinely offered in public or private hearing healthcare sectors in the UK.

In summary, there is some low quality evidence that ACE and similar programmes improve communication function and quality-of-life and these outcomes may be enhanced when delivered in conjunction with a hearing aid. Communication programmes involve a substantial commitment on the part of participants and those who run and pay for them. Recent evidence shows that it is effective and feasible to deliver ACE as an alternative intervention in Australia and Sweden.[43 44 49 50] There is now a need to establish whether reported clinical and economic benefits of ACE and communication programmes like ACE can be achieved in the context of NHS hearing aid provision. This protocol for The ACE To HEAR study (**A**ctive **C**ommunication **E**ducation **To** improve **HEAR**ing) is intended to deliver ACE to unsuccessful or struggling new NHS hearing aid users, three-months post-fitting, in order to assess whether a large RCT designed to evaluate the effectiveness of ACE in improving hearing aid benefit within the UK National Health Service (NHS) is feasible.

METHODS AND ANALYSIS

This protocol was developed and is reported according to the SPIRIT (Standard Protocol Items for Randomized Trials) statement.[51]

Study Aim:

The aim of this study is to determine the feasibility of delivering a future, full-scale randomised controlled trial (RCT) to evaluate ACE plus treatment-as-usual versus treatment-as-usual alone, within the UK National

For peer review only - http://bmjopen.bmj.com/site/about/guidelines.xhtml

Health Service (NHS) in two UK cities. Treatment-as-usual is defined as a referral from a patient's GP to audiology services to treat permanent hearing loss. It comprises up to two appointments for hearing aid fitting and a third face to face or telephone follow-up appointment.

Study objectives

Objectives will evaluate ACE delivery and trial delivery processes.

ACE Delivery Objectives

1) To assess ACE up-take rates, eligibility and acceptability of clinic location (between and within the two study sites) for participants and SOs.
2) To evaluate the level of ACE attendance and retention amongst participants randomised to the ACE arm of the study.
3) To assess acceptability of ACE with participants, SOs and audiologists.
4) To assess capability, capacity and willingness of audiology departments to support delivery of ACE within existing services.
5) To assess intervention fidelity of delivering ACE.

Trial Delivery Objectives

6) To assess RCT recruitment rates, evaluate the randomisation process and time to accrue ACE groups.
7) To assess the acceptability of study processes to participants, SOs and audiologists (i.e. those related to recruitment, the feasibility of identifying struggling hearing aid users, randomisation process, data collection, measurement of ACE fidelity and acceptability)
8) To explore patient reported outcome measures (PROMs) and estimate likely standard deviations, including quality-of-life tools (EQ-5D-5L; SF-36) and a bespoke health care resource use/acceptability/utility questionnaire for use in an intended full-scale RCT.

Study design

For peer review only

For peer review only - http://bmjopen.bmj.com/site/about/guidelines.xhtml

The study design will be a pragmatic, randomised controlled, open feasibility trial with embedded economic and process evaluations delivered in one audiology department in each of two UK cities. The design of the trial is shown in Figure 1.

Study setting

Study sites for this feasibility study are the Audiology Departments at York Hospital (YH), York Teaching Hospitals NHS foundation Trust, and the Bradford Royal Infirmary (BRI), Bradford Teaching Hospitals NHS Foundation Trust.

Study population

The study population will consist of adult patients aged 18 years or over, receiving treatment-as-usual delivered in one of the two participating centres. They will be considered potentially eligible if all of the following eligibility criteria apply at their 3-month post-fitting follow-up appointment:

Inclusion criteria:

a) Moderate or less than moderate benefit, defined by IOI-HA question 2.[52 53]
b) Hearing impairment: pure-tone average better ear thresholds at 500, 1000, 2000, and 4000 Hz of more than 25 dB HTL.
c) No significant self-reported history of neurological impairment.
d) Willing to provide written informed consent.
e) Able to provide written informed consent.
f) Able to take part in the intervention by understanding and using spoken English.
g) Able to self-complete the English language outcome measure tools.
h) The following inclusion criteria for Significant Others (SOs) will be assessed:
    a. A spouse or other family member who lives with or is a carer for a patient recruited to the study.

Exclusion criteria:

For peer review only - http://bmjopen.bmj.com/site/about/guidelines.xhtml

a) Severe or profound bilateral hearing impairment. Pure-tone better ear average thresholds measured at 500, 1000, 2000, and 4000 Hz of more than 85 dB HTL.

b) Significant on-going ear related health or mental health issues that, in the audiologist's or associate audiologist's professional opinion would preclude hearing aid fitting or attendance at ACE sessions.

c) Unable or unwilling to give written informed consent.

In addition, SOs will be excluded if they are unable or unwilling to give written informed consent.

Patients who do not have a SO or family member able to attend the ACE sessions are still eligible to participate in the study.

Sample size calculation and recruitment

As this is a feasibility study, the main purpose is to assess the acceptability and feasibility of conducting this study, with a view to designing and conducting a future full-scale trial.[54] Six ACE groups are planned with up to seven patients in each (five minimum). This leads to a maximum sample size of 44 patients and up to 44 significant others for the ACE intervention arm, and 44 in the control arm (n=88). This sample size will allow the standard deviation of the proposed outcome measures to be estimated with reasonable certainty to inform future sample size calculations.[54] Collectively the two study sites fit approximately 4300 hearing aids a year and based on their experience we estimate 10% will require extra help at follow-up and be eligible for inclusion. Recruitment of patients commenced on the 1st April 2017 and is now underway. The recruitment window is currently planned to end on 28th February 2018 and there is potential to extend this phase until 30th April 2018 if necessary.

Randomisation

Eligible, consenting patients from the same study site who have completed baseline assessments will be randomised by a remote, centralised randomisation service (provided by York Trials Unit) in batches of 10-14 (intervention: control ratio of 1:1) using block randomisation in a single large block per batch. Following randomisation, a letter outlining the next steps will be sent to participants. For those allocated to the ACE

For peer review only - http://bmjopen.bmj.com/site/about/guidelines.xhtml

arm, this will contain an invitation for them and their SO to attend five ACE sessions, including details of the venue, times and information.

Blinding

By the nature of the interventions used within this study, blinding of the participants and facilitator is not possible. The research team responsible for data analysis and reporting will be blinded where possible.

Intervention allocation

Consenting participants will be randomly allocated to either:

a) **Treatment-as-usual:** Participants randomised to receive Treatment-as-usual will continue to receive usual care delivered by their service.

b) **ACE plus Treatment-as-usual**: Participants randomised to receive the Active Communication Education (ACE) plus Treatment-as-usual, will attend five two hour weekly sessions of the ACE programme, developed in Australia [43] and will continue to receive usual care delivered by their service.

The ACE programme will be delivered as described in the published ACE manual[55] by a trained facilitator to groups of hard of hearing people and SOs where possible. The same audiologist facilitator will deliver ACE to all groups.

ACE consists of six modules based on everyday communication activities known to be problematic for hard of hearing adults: Module 1: Communication needs analysis; Module 2: Conversation in background noise; Module 3: Conversation around the house; Module 4: Communication with difficult speakers; Module 5: Listening to other signals; and Module 6: Listening to public-address systems.

Through the use of demonstrations, practical exercises, discussion and problem solving, participants and significant others will learn individual problem-solving skills which can be applied in a range of novel situations and discuss the use of communication strategies, lip reading skills, clarification skills, and assistive technology.

For peer review only - http://bmjopen.bmj.com/site/about/guidelines.xhtml

Intervention fidelity

Assessment of the fidelity to the ACE training of the audiologist facilitator and the delivery of the ACE programme will follow guidance from the NIH Behaviour Change Consortium, [61] and conducted in a pragmatic approach mirroring the way fidelity of an educational programme, such as ACE, would be realistically assured in an NHS context. The trainers will reflect on the training sessions; fidelity of ACE delivery will be *facilitated* by supervision of the audiologist for the first session, with feedback and self-reflection used to address any issues; fidelity of ACE delivery will then be *assessed* via a self-monitoring form recording on a 1-4 scale (poor – good) of their adherence to the ACE manual for each module across all sessions.

Intervention Compliance

Measuring compliance is challenging in complex interventions such as this, where there are a number of interacting elements (such as the influences of the ACE facilitator, participants' significant other, a varied selection of ACE module topics worked on and each individuals' perceptions of their (or their SO's) disability and treatment benefit). The intervention to be delivered is defined in the ACE manual[55] and our understanding (measurement) of what is actually delivered will be informed by the fidelity measures above, weekly attendance logs and weekly session records. ACE participants and SOs will self-report goal setting for each module and the number of goals achieved during the programme, the number of completed exercises (homework) will be reported providing an additional measure of the level of engagement or compliance with the programme that is focused on the participant and their SO.

Concurrent treatments

Any additional treatments identified will be available to both arms when necessary (e.g. hearing aid repairs, replacement batteries, etc.). The bespoke resource use questionnaire (see below) will allow us to monitor additional treatment accessed during the study.

For peer review only - http://bmjopen.bmj.com/site/about/guidelines.xhtml

Outcome Measures

Figure one shows the main processes of the trial, their relationship with the outcome measures and their timing. Data will be collected at baseline (prior to randomisation); during all ACE sessions and after ACE session 5 for ACE participants; and at an equivalent time for Treatment-as-usual participants; and approximately 3 months post-randomisation (6 months post-hearing aid fitting).

The feasibility and the potential for a future large-scale study design (the study's aim) will be measured by recording and evaluating:

ACE Delivery Outcomes:

1. Attendance: Attendance rates of participants and SOs at ACE sessions will be measured and who attends ACE session with the participant will be recorded (objective 2).
2. Fit of ACE with existing variations in service delivery models: Comparison of attendance at ACE delivered at different study sites and their satellite clinics and the effect of using telephone or face-to-face follow-up appointments will be recorded (objectives 1 and 4).
3. Can ACE be delivered as intended in the ACE protocol? This will be assessed through: (i) time taken to train the ACE facilitator successfully; (ii) number of ACE goals achieved by participants; and (iii) facilitator's adherence to the ACE protocol (fidelity) (objective 5).
4. Acceptability: Bespoke acceptability questionnaire regarding study processes, designed to explore participant and SO thoughts regarding the study including; ACE session organisation, session content, being approached and informed about the study and completing the questionnaires. The questionnaire is administered to participants and SOs at final ACE session and at a comparable time for the questions for Treatment-as-usual arm (objectives 3 and 4).

RCT Delivery Outcomes:

For peer review only - http://bmjopen.bmj.com/site/about/guidelines.xhtml

5. Recruitment: Number of follow-up cases in GP referred pathway; number of follow-up cases in GP referred pathway struggling with their hearing aid; number of and reasons for exclusions; number of patients who decline to participate and reason for declining; number who miss ACE intervention window (i.e. unable to attend an ACE group within 1-3 weeks after randomisation); number given an appointment for an ACE group session; and number of consented participants who fail to attend ACE sessions (objectives 6 and 7).
6. Allocation: Time taken to recruit and logistics of recruiting an optimally sized and located ACE group; time ACE started after randomisation (ACE intervention window); (objective 6).
7. Patient Reported Outcome Measure Data: Completion of the outcome measures below at each time-point will be recorded as well as extent of missing data within each outcome measure (objective 8)

Patient Reported Outcome Measures:

- International Outcomes Inventory for Hearing Aids (IOI-HA): [52] a seven-item questionnaire designed to evaluate the effectiveness of hearing aid treatments. The domains covered are: daily use; benefit; residual activity limitations; satisfaction; residual participation restrictions; impact on others; and quality of life. Responses are assigned a value from 1 to 5 and values summed. Higher scores indicate a more favourable outcome.
- International Outcomes Inventory for Alternative Interventions (IOI-AI): [56] a version of the IOI designed for use for non-hearing aid based interventions such as ACE, covering the same domains as the IOI-HA.
- Self-Assessment of Communication (SAC):[57] designed to measure the effect of hearing loss and hearing aid outcomes. This ten-item instrument covers questions about communication problems using a Likert scale ranging from 1 ("almost never") to 5 ("practically always"). A percentage score is calculated by multiplying the raw score by 2, subtracting 20, and multiplying by 1.25.

For peer review only - http://bmjopen.bmj.com/site/about/guidelines.xhtml

- EQ-5D-5L:[58 59] A standardised generic instrument for describing and valuing health in terms of five dimensions (Mobility, Self-Care, Usual Activities, Pain/Discomfort, and Anxiety/Depression) using five levels of severity. Overall health on the day is also rated by the respondent on a 0–100 vertical visual analogue scale (EQ-VAS).
- Short-Form 36 (SF-36):[60] a generic health measure with 36 items assessing eight health concepts: physical functioning; role limitations due to physical problems; general health perceptions; vitality; social functioning; role limitations due to emotional problems; general mental health; and health transition

The following will be completed by participants' SOs only:

- International Outcome Inventory for Alternative Interventions: version for Significant Others (IOI-AI-SO):[56] a version of the IOI designed for use with SOs and non-hearing aid based interventions covering the same seven domains as the IOI-HA.
- International Outcomes Inventory for Hearing Aids: version for Significant Others (IOI-HA-SO):[56] an extension of the IOI-HA for use with the SO covering the same seven domains as the IOI-HA.
- Significant Other Scale for Hearing Disability (SOS-HEAR):[16] a 27-item self-report tool, which assesses third-party disability in spouses of older people with hearing impairment. It measures the effects of hearing impairment on the SO in the following domains: Changes to communication; Communication burden; Relationship changes; Going out and socializing; Emotional reactions to adaptations; Concern for partner. It uses a 5-point response scale: 0= no problem, to 4 = a complete problem.

The feasibility of collecting postal questionnaire data at each time point will be evaluated. Table 1 shows the data collection schedule.

For peer review only - http://bmjopen.bmj.com/site/about/guidelines.xhtml

Table 1: Data collection schedule: data are collected approximately three months post hearing aid fitting (Baseline); at each ACE session 1-5 for the intervention arm and at a time equivalent to ACE week 5 for the Treatment-as-usual arm; and approximately six months post hearing aid fitting. ~ ACE arm only. * Significant Others only

Study period:	Recruitment	Allocation	Post allocation					
Time point:	Baseline	0	ACE week 1	ACE week 2	ACE week 3	ACE week 4	ACE week 5	6 month
**RECRUITMENT:**								
Eligibility	•							
Informed consent	•							
Optional qualitative study consent	•							
Allocation		•						
**ASSESSMENTS:**								
Demographics	•							
IOI-HA	•						•	•
SAC	•							•
EQ-5D-5L	•							•
SF36	•							•
Resource use	•							•
IOI-AI~							•	•
ACE Participant attendance~			•	•	•	•	•	
ACE SO attendance~			•	•	•	•	•	
IOI-AI-SO~*							•	•
IOI-HA-SO*	•						•	•
SOS-HEAR*	•						•	•
Acceptability questionnaire							•	
Qualitative interviews (Participant & SO)							•	
Qualitative interviews (Audiologists)								•

For peer review only - http://bmjopen.bmj.com/site/about/guidelines.xhtml

Screening and enrolment

Patients attending Audiology Clinics at York Hospital (YH) and Bradford Royal Infirmary (BRI) will be approached to take part. Treatment-as-usual provided at the post hearing aid fitting follow-up will be according to site and therefore the recruitment process will vary slightly at each site:

a) York: patients will be followed up via a telephone interview. Eligibility will be checked during telephone interview and from medical records. Details of eligible and interested patients will, with their permission, be passed onto a non-ACE researcher who will post out a PIS and conduct a telephone follow-up call a few days later to see if still interested. Contact information is provided in the PIS so that the patient has opportunity to ask questions regarding the study. If willing to participate, informed consent and baseline questionnaire will be completed by post. SOs of patients who are recruited at York will receive a SO-specific PIS, consent form and baseline questionnaire by post to be returned in a freepost envelope.

b) Bradford: patients will be offered a face-to-face follow-up appointment 3-months post hearing aid fitting. Eligibility will be checked at this appointment and from medical records. Details of eligible and interested patients will be passed onto a non-ACE researcher who will provide a patient information sheet (PIS) and discuss the study. The patient will have the opportunity to ask questions and if willing, provide informed consent and complete a baseline questionnaire. If further consideration is required, the patient will be contacted by telephone call a few days later to see if still interested. In addition, if an SO attends the appointment with a patient, they will be provided with a SO-specific PIS, consent form and baseline questionnaire. Otherwise the documentation will be given to the patient to pass on to their SO or posted out.

We will monitor the proportion of patients referred to the Treatment-as-usual pathway during the study recruitment who subsequently do not attend or are not contactable by telephone for their post HA fitting

For peer review only - http://bmjopen.bmj.com/site/about/guidelines.xhtml

follow-up in order to estimate how many referrals may potentially be lost to recruitment. We will liaise with audiologists to identify reasons for non-attendance where possible.

Data collection and management

All data for the participant outcome measures will be collected by self-completed questionnaires returned by post or in secure boxes within the audiology clinics. Participants and SOs who fail to return their postal questionnaires will receive one reminder letter. Participants may also be asked to complete a questionnaire over the telephone, or asked to provide any missing data if required. Data from these paper forms will then be entered into a master database for the trial using either optical scanning techniques or entered manually.

Participants may withdraw from all or any aspects of the study without influencing their future care or treatment. A brief update of how the study is progressing will be sent out in order to maintain participant engagement with the study.

All information collected during the course of the trial will be kept strictly confidential. Information will be held securely on paper and electronically at York Trials Unit. All trial data will be identified using a unique trial identification number. Analytical datasets will not contain any identifiable information. Data will be archived for a period of at least 10 years following the end of the study.

Statistical analysis

A single analysis will be conducted at the end of the trial using Stata v13 or later. Data summaries and analyses will inform the design of a full-scale RCT of the intervention. Baseline data will be summarised by trial arm, using descriptive statistics for continuous data (mean, standard deviation (SD), median, minimum, maximum, number missing) and counts and percentages for categorical data. Recruitment rates will be reported monthly and overall, and by site. The flow of participants through the trial will be detailed in a CONSORT flow diagram and referral, consent and attendance rates will be summarised overall and by site using counts, percentages and 95% confidence intervals.

For peer review only

For peer review only - http://bmjopen.bmj.com/site/about/guidelines.xhtml

The number of ACE sessions attended will be summarised alongside any SOs who attended the sessions. Summaries will be provided overall, by site/clinic and by whether follow-up appointments were made as single or block booking. Acceptability data using Likert scales at 6 months, for participants, SOs and audiologists will be summarised separately using summary statistics and presented graphically using bar charts, by trial arm.

The number of participants withdrawing from the ACE intervention and/or the trial and any reasons for withdrawal will be summarised.

The time taken to train audiologists to deliver ACE and the number of ACE goals achieved by participants will be summarised. Fidelity scores (from self-monitoring forms) will be summarised overall and by session. The proportion of training and ACE intervention delivered as intended, as well as any adaptations to training/intervention will be reported. Variations in dose of ACE intervention will be measured through ACE attendance and attrition data.

Questionnaire return rates at each time point will be presented overall and by trial arm. PROMs at each time point will be summarised descriptively overall, by trial arm, and by ACE group for participants allocated to receive the ACE intervention. Standard deviations will be presented with 80% confidence intervals to inform future sample size calculations. The proportion of participants at the floor and ceiling of each measure, at each time point, will be reported along with the standardised response mean (SRM) to measure the sensitivity of each questionnaire to detect change. The SRM will be calculated as mean change in scores or values divided by the standard deviation in change scores.[63] Questionnaire completion times (from self-report) will be summarised as a consideration for instruments going into the full-scale evaluation.

Qualitative data

Following delivery of all the ACE intervention sessions:

For peer review only - http://bmjopen.bmj.com/site/about/guidelines.xhtml

- The facilitator will be interviewed to explore the training and implementation process and their experiences of delivering ACE including barriers/facilitators to adhering to the ACE protocol.
- Up to three audiologists from both study sites (up to six in total) will also be interviewed, exploring the capability, capacity and willingness of their audiology departments to support the ACE study within their existing services. The acceptability of study processes will also be explored.
- A sample of 10-12 participants in the ACE intervention arm and four participants in the control arm (with their SOs if willing) will also be interviewed as soon as possible after the completion of the ACE sessions. We will purposively select participants to ensure a mix of those with good/poor hearing aid outcomes (measured at the 5th and final ACE session and equivalent control arm time point, see Fig 1. for outcome measures) as well as ensuring we include some participants who dropped out of the sessions/study, ensuring a wide range of views are collected. Control arm participants will include those who have dropped out from the study where possible, allowing us to explore reasons for this. SOs will be interviewed as part of a dyad with the participant. Semi-structured interviews will explore the acceptability of the ACE (e.g. venue, timing, content), its perceived impact (reflecting on hearing aid outcomes) with ACE intervention arm participants; and views on study processes (e.g. recruitment, outcome measures and timing) with ACE intervention and control arm participants.

Interviews will be audio-recorded, transcribed verbatim and analysed (with NVivo-11) using the Framework approach[62] which is particularly useful for analysing qualitative data in a pragmatic yet systematic way, where theoretical development is not needed. The steps are familiarisation, construction of a thematic framework, indexing and charting the data, mapping and interpretation.

Economic analysis and quality of life data

A full cost-effectiveness analysis will not be conducted as this is a feasibility trial thus the study is not powered to detect significant differences.

For peer review only

The costs of implementing the ACE intervention will be estimated and the potential resource implications versus usual care will be explored. Resource use will be summarised by resource use type (e.g. GP appointments, outpatient appointments) and appropriate unit costs to be applied to each resource use type will be identified. These will be sourced from a combination of local costings and national databases.[64] The costing approach will take a broad analytical perspective accounting not only for NHS costs but also for those observed by patients, though this cost will be presented separately. It is anticipated that additional resources utilised in the ACE intervention arm will largely be NHS staff time and travel/time for patients.

A reliable and valid tool to capture resource use will be developed and tested during the feasibility trial to ensure that all necessary data for a full economic evaluation can be captured.

Methods to estimate an incremental cost-effectiveness ratio for the ACE intervention versus Treatment-as-usual alone in terms of Quality Adjusted Life Years (QALYs) will be explored. No health-related quality of life assessment tool is currently sufficiently sensitive to all populations, and in the field of hearing health, there has been limited research to identify the most effective tool. In the UK, NICE advocates the used of the EQ-5D for generating quality adjusted life years, though it is acknowledged that this is not always the most sensitive tool for particular populations for whom the majority of its dimensions may not apply.[65] In the US analysis of a four week rehabilitation programme noted above,[48] the SF-36 was used to generate QALYs rather than the EQ-5D, with the SF-36 showing a reduction in the cost per QALY in favour of the intervention. For the present feasibility trial, both assessments will be used to elicit QALYs and a comparison will be made between the outcomes of the two measures. This will enable a decision to be made as to which tool would be most appropriate in the full-scale trial.

The feasibility work will also be used to help to identify any patterns of missing data and any issues relevant for sensitivity analysis which will influence statistical plans for dealing with imprecision and other uncertainties in the full RCT. For example, data can be bootstrapped to account for the expected skewness

For peer review only - http://bmjopen.bmj.com/site/about/guidelines.xhtml

evident in economic cost data. The data collected as part of this feasibility study will be used to inform subsequent pre-trial modelling.

Adverse events

Risks within this study are considered to be minimal. It is considered highly unlikely that the ACE intervention arm will suffer any adverse consequences as a result of receiving the ACE plus Treatment-as-usual. Nevertheless, interviews with ACE participants, the ACE facilitator and ACE questionnaire data will be used to monitor this eventuality.

Trial monitoring and oversight

Due to the low risk nature of this trial, approval has been obtained to set up one independent steering and monitoring committee to undertake the roles traditionally undertaken by the Trial Steering Committee and Data Monitoring and Ethics Committee. Regular meetings of a Study Management Group will take place to oversee the progress of the study and review recruitment. We will establish a Project Advisory Panel (PAP) with between two and four hard of hearing adults or hearing spouses that will meet a minimum of five times over the duration of the project.

Data Monitoring and management

Information relating to study participants will be kept confidential and managed in accordance with the Data Protection Act, NHS Caldicott Guardian, Research Governance Framework for Health and Social Care and the Research Ethics Committee (REC) approval.

Participant details will be stored on a secure password protected server located at the University of York, for the purposes of assisting in follow-ups during the study. All paper data collected from participants will be maintained in a safe secure environment at York Trials Unit. Paper records will be identified using identifiers rather than personally identifiable information. Analytical datasets will not contain any identifiable information.

For peer review only - http://bmjopen.bmj.com/site/about/guidelines.xhtml

The confidentiality of the participants, SOs and audiologists interviewed during their qualitative interviews will be ensured by assigning a unique identification code to electronic sound files and transcripts of interviews, known only to the qualitative researcher and appropriate members of the research team. Any quotes published will be anonymous further protecting participant confidentiality.

Amendments

Since the study started in February 2017, three HRA approved amendments were added to the protocol and are included in the final version reported here:

1. Revised the fidelity check tool. This was considered a non-substantial amendment.
2. Remove inclusion criteria of less than three hours hearing aid use a day and adjusted inclusion criteria b) to include moderate benefit. We also gained approval to distribute study information flyers to study site staff and patients. These were considered substantial amendments.
3. Developed a study information flyer to be sent to patients not contactable by telephone for follow-up at the York study site. This was considered a non-substantial amendment.

ETHICS AND DISSEMINATION

Ethical approval has been granted by South East Coast—Surrey Research Ethics Committee (16/LO/2012). The proposed study will be conducted in accordance with the MRC Guidelines on Good Clinical Practice in Clinical Trials.

The results from this study will be submitted to the funders, peer-reviewed journals, presented at relevant meetings/conferences and within the participating and other audiology departments. We also intend to present the findings of this study in patient forums, Trust bulletins and PPI activities including newsletters and public interest groups who work and support older adults with hearing impairment.

CONCLUSION

For peer review only - http://bmjopen.bmj.com/site/about/guidelines.xhtml

This will be the first RCT of this type of group communication programme in the context of a public health service and as an adjunct to hearing aid fitting. The impact of this study will ultimately be realised by a larger fully powered RCT designed to determine the effectiveness of the ACE intervention in improving hearing aid benefit for hearing aid users within the NHS GP referral for a hearing aid pathway in the UK. The primary and secondary objectives of this study will inform such a RCT.

For peer review only

For peer review only - http://bmjopen.bmj.com/site/about/guidelines.xhtml

FIGURE CAPTIONS

Figure 1: ACE To HEAR Study flow diagram. The diagram was developed using CONSORT guidelines (http://www.consort-statement.org/) and indicates the main processes of the trial, their relationship with the outcome measures and their timing.

For peer review only

For peer review only - http://bmjopen.bmj.com/site/about/guidelines.xhtml

REFERENCES

1. Organisation WH. WHO global estimates on prevalence of hearing loss: Mortality and Burden of Diseases and Prevention of Blindness and Deafness. Secondary WHO global estimates on prevalence of hearing loss: Mortality and Burden of Diseases and Prevention of Blindness and Deafness. 2012. http://www.who.int/pbd/deafness/WHO_GE_HL.pdf.
2. Collins JG. Prevalence of selected chronic conditions: United States 1990-1992. Vital Health Stat 10(194) 1997.
3. Davis AC. The Prevalence of Hearing Impairmrent and Reported Hearing Disability Among Adults in Great Britain. International Journal of Epidemiology 1989;**18**(4):911-17.
4. AoHL. Hearing Matters: Taking action on hearing loss in the 21st century. http://www.actiononhearingloss.org.uk/supporting-you/policy-research-and-influencing/research/hearing-matters.aspx: Action on Hearing Loss, 2011.
5. Roth TN, Hanebuth D, Probst R. Prevalence of age-related hearing loss in Europe: a review. European Archives of Oto-Rhino-Laryngology 2011;**268**(8):1101-07.
6. Pronk M, Kramer SE, Davis AC, et al. Interventions following hearing screening in adults: A systematic descriptive review. International Journal of Audiology 2011;**50**(9):594-609.
7. Bess FH, Lichtenstein MJ, Logan SA, et al. Hearing impairment as a determinant of function in the elderly. J Am Geriatr Soc 1989;**37**(11):123-28.
8. Chia EM, Wang JJ, Rochtchina E, et al. Hearing impairment and health-related quality of life: The blue mountains hearing study. Ear and Hearing 2007;**28**:187-95.
9. Health Quality Ontario. Social isolation in community-dwelling seniors: an evidence-based analysis. Ontario health technology assessment series 2008;**8**(5):1-49.
10. Gopinath B, Schneider J, Hickson L, et al. Hearing handicap, rather than measured hearing impairment, predicts poorer quality of life over 10 years in older adults. Maturitas 2012;**72**(2):146-51.

For peer review only

For peer review only - http://bmjopen.bmj.com/site/about/guidelines.xhtml

11. Hetu R, Jones L, Getty L. The impact of acquired hearing impairment on intimate-relationships - implications for rehabilitation Audiology 1993;**32**(6):363-81.

12. Heine C, Browning C. The communication and psychosocial perceptions of older adults with sensory loss: a qualitative study. Ageing and Society 2004;**24**:113-30.

13. Heine C, Browning CJ. Communication and psychosocial consequences of sensory loss in older adults: overview and rehabilitation directions. Disability and Rehabilitation 2002;**24**(15):763-73.

14. Humes LE, Dubno JR, Gordon-Salant S, et al. Central Presbycusis: A Review and Evaluation of the Evidence. Journal of the American Academy of Audiology 2012;**23**(8):635-66.

15. Stephens D, Kramer SE. Living with hearing loss: The Process of Enablement. . London: Wiley, 2009.

16. Scarinci N, Worrall L, Hickson L. The effect of hearing impairment in older people on the spouse: Development and psychometric testing of The Significant Other Scale for Hearing Disability (SOS-HEAR). International Journal of Audiology 2009;**48**(10):671-83.

17. Wallhagen MI, Strawbridge WJ, Shema SJ, et al. Impact of self-assessed hearing loss on a spouse: A longitudinal analysis of couples. Journals of Gerontology Series B-Psychological Sciences and Social Sciences 2004;**59**(3):S190-S96.

18. Scarinci N, Worrall L, Hickson L. Factors Associated With Third-Party Disability in Spouses of Older People With Hearing Impairment. Ear and Hearing 2012;**33**(6):698-708.

19. Kochkin S. MarkeTrak VIII patients report improved quality of life with hearing aid usage. Hearing Journal 2011;**64**(6):25-32.

20. Laplante-Levesque A, Hickson L, Worrall L. Rehabilitation of Older Adults With Hearing Impairment: A Critical Review. Journal of Aging and Health 2010;**22**(2):143-53.

21. Ferguson MA, Kitterick PT, Chong LY, et al. Hearing aids for mild to moderate hearing loss in adults. Cochrane Database of Systematic Reviews 2017(9).

For peer review only - http://bmjopen.bmj.com/site/about/guidelines.xhtml

22. RNID. Royal National Institute for the Deaf. Information and resources. Secondary Royal National Institute for the Deaf. Information and resources 2011. http://www.rnid.org.uk/information_resources/

23. Davis A, Smith P, Ferguson M, et al. Acceptability, benefit and costs of early screening for hearing disability: a study of potential screening tests and models. Health Technology Assessment 2007;**11**(42).

24. Gianopoulos I, Stephens D, Davis A. Follow up of people fitted with hearing aids after adult hearing screening: the need for support after fitting. BMJ 2002;**325**.

25. Hougaard S, Ruf S. EuroTrak I: A consumer survey about hearing aids in Germany, France and the UK. First global comparative study of hearing, hearing loss, and hearing aids. The Hearing Review 2011;**18**(2):12-28.

26. Stephens D, Lewis P, Davis A, et al. Hearing aid possession in the population: Lessons from a small country. Audiology 2001;**40**(2):104-11.

27. Lupsakko TA, Kautiainen JH, Sulkava R. The non-use of hearing aids in people aged 75 years and over in the city of Kuopio in Finland. European Archives of Oto-Rhino-Laryngology 2005;**262**:165-69.

28. Popelka MM, Cruickshanks KJ, Wiley TL, et al. Low prevalence of hearing aid use among older adults with hearing loss: The epidemiology of hearing loss study. Journal of the American Geriatrics Society 1998;**46**(9):1075-78.

29. Smeeth L, Fletcher AE, Siu-Woon Ng E, et al. Reduced hearing, ownership, and use of hearing aids in elderly people in the UK-the MRC Trial of the Assessment and Management of Older People in the Community: a cross-sectional survey. The Lancet 2002;**359**(9316):1466-70.

30. Department of Health. Modernising NHS hearing aid services London: Department of Health, 2000.

31. Barker F, Mackenzie E, Elliott L, et al. Interventions to improve hearing aid use in adult auditory rehabilitation (Review). Cochrane Database of Systematic Reviews 2016; **Art. No.: CD010342**(8).

32. Ng JH-Y, Loke AY. Determinants of hearing aid adoption and use among the elderly: A systematic review. International Journal of Audiology 2015;**54**(5):291-300.

For peer review only

33. Wallhagen MI. The Stigma of Hearing Loss. Gerontologist 2010;**50**(1):66-75.

34. Laplante-Levesque A, Hickson L, Worrall L. What Makes Adults With Hearing Impairment Take Up Hearing Aids or Communication Programs and Achieve Successful Outcomes? Ear and Hearing 2012;**33**(1):79-93.

35. Laplante-Lévesque A, Hickson L, Worrall L. Factors influencing rehabilitation decisions of adults with acquired hearing impairment. International Journal of Audiology 2010;**49**(7):497-507.

36. Laplante-Levesque A, Knudsen LV, Preminger JE, et al. Hearing help-seeking and rehabilitation: Perspectives of adults with hearing impairment. International Journal of Audiology 2012;**51**(2):93-102.

37. Southall K, Gagne JP, Leroux T. Factors that influence the use of assistance technologies by older adults who have a hearing loss. International Journal of Audiology 2006;**45**(4):252-59.

38. Scarinci N, Worrall L, Hickson L. The effect of hearing impairment in older people on the spouse. International Journal of Audiology 2008;**47**(3):141-51.

39. Scarinci N, Worrall L, Hickson L. The ICF and third-party disability: Its application to spouses of older people with hearing impairment. Disability and Rehabilitation 2009;**31**(25):2088-100.

40. Gagne JP, Jennings MB, Southall K. The ICF: A Classification System and Conceptual Framework Ideal for Audiological Rehabilitation. Perspectves on Aural Rehabilitation and its Instrumentation 2009;**16**(1):8-14.

41. Lockey K, Jennings MB, Shaw L. Exploring hearing aid use in older women through narratives. International Journal of Audiology 2010;**49**(8):542-49.

42. WHO. ICF: International Classification of Fuctioning, Disability and Health. Geneva: World Health Organisation, 2001.

43. Hickson L, Worrall L, Scarinci N. A randomized control trial evaluating the Active Communication Education program for older people with hearing impairment. Ear and Hearing 2007;**28**(2):212-30.

For peer review only - http://bmjopen.bmj.com/site/about/guidelines.xhtml

44. Oberg M, Bohn T, Larsson U. Short- and Long-Term Effects of the Modified Swedish Version of the Active Communication Education (ACE) Program for Adults with Hearing Loss. Journal of the American Academy of Audiology 2014;**25**(9):848-58.

45. Hawkins DB. Effectiveness of counseling-based adult group aural rehabilitation programs: a systematic review of the evidence. Journal of the American Academy of Audiology 2005;**16**(7):485-93.

46. Chisolm TH, Abrams HB, McArdle R. Short- and long-term outcomes of adult audiological rehabilitation. Ear and Hearing 2004;**25**(5):464-77.

47. Preminger JE. Issues associated with the measurement of psychosocial benefits of group audiologic rehabilitation programs. Trends Amplif 2007;**11**(2):113-23.

48. Abrams H, Chisolm TH, McArdle R. A cost-utility analysis of adult group audiologic rehabilitation: Are the benefits worth the cost? Journal of Rehabilitation Research and Development 2002;**39**(5):549-57.

49. Hickson L, Worrall L. Beyond hearing aid fitting: improving communication for older adults. International Journal of Audiology 2003;**42**:S84-S91.

50. Oberg M, Bohn T, Larsson U, et al. A Preliminary Evaluation of the Active Communication Education Program in a Sample of 87-Year-Old Hearing Impaired Individuals. Journal of the American Academy of Audiology 2014;**25**(2):219-28.

51. Chan A, Tetzlaff JM, Altman DG, et al. Spirit 2013 statement: Defining standard protocol items for clinical trials. Annals of Internal Medicine 2013;**158**(3):200-07.

52. Cox RM, Alexander GC. The International Outcome Inventory for Hearing Aids (IOI-HA): psychometric properties of the English version. International Journal of Audiology 2002;**41**(1):30-35.

53. Cox RM, Alexander GC, Beyer CM. Norms for the international outcome inventory for hearing aids. Journal of the American Academy of Audiology 2003;**14**(8):403-13.

54. Teare M, Dimairo M, Shephard N, et al. Sample size requirements to estimate key design parameters from external pilot randomised controlled trials: a simulation study. Trials 2014;**15**(1):264.

For peer review only

For peer review only - http://bmjopen.bmj.com/site/about/guidelines.xhtml

55. Hickson L, Worrall L, Scarinci N, editors. *ACE: Active Communication Education.* : University of Queensland, 2015.

56. Noble W. Extending the IOI to significant others and to non-hearing aid-based interventions. International Journal of Audiology 2002;**41**(1):27-29.

57. Schow RL, Nerbonne MA. communication screening profile - use with elderly clients. Ear and Hearing 1982;**3**(3):135-47.

58. Herdman M, Gudex C, Lloyd A, et al. Development and preliminary testing of the new five-level version of EQ-5D (EQ-5D-5L). Quality of Life Research. Quality of Life Research 2011;**20**(10):1727-36.

59. Janssen M, Pickard A, Golicki D, et al. Measurement properties of the EQ-5D-5L compared to the EQ-5D-3L across eight patient groups: a multi-country study. Quality of Life Research 2013;**22**(7):1717-27.

60. Ware J, Sherbourne C. The MOS 36-item short-form health survey (SF-36) 1: conceptual framework and item selection. Med Care 1992;**30**:473-83.

61. Bellg AJ, Resnick B, Minicucci DS, et al. Enhancing Treatment Fidelity in Health Behavior Change Studies: Best Practices and Recommendations From the NIH Behavior Change Consortium. Health Psychology 2004;**23**(5):441-51.

62. Ritchie J, Lewis J. *Qualitative Research Practice*. London: Sage Publications, 2003.

63. Stratford PW, Binkley J, Solomon P, et al. Defining the minimum level of detectable change for the Roland-Morris questionnaire. Physical Therapy 1996;**76**(4):359-65.

64. Curtis L. Unit Costs of Health and Social Care 2014, 2014.

65. NICE. Guide to the Methods of Technology Appraisal.: National Institute of Health and Clinical Excellence: London, 2013.

For peer review only

For peer review only - http://bmjopen.bmj.com/site/about/guidelines.xhtml

Contributorship Statement

NJT led on the conception, design and writing of the study and study protocol with substantial contributions to the design, writing, critical review of intellectual content and final manuscript approval from JW, CJ, KC, AF, LH, LC, KB, CF, CM,RG, KI and LG. All authors agree to be accountable for their work. As Principle Investigator, NJT takes overall responsibility for the work.

KC provided statistical expertise in the study design and development stages of the project and the protocol. CF provided further essential statistical advice and expertise on the study protocol.

JW, CJ and LC made substantial contributions to the trial design and management. AF was involved in all aspects of the study and LG provided particular input to PPI. KB was specifically responsible for the health economic aspects of the study design and KI and RG were responsible for aspects specific to Trust's service delivery, providing expert clinical support.

Data Sharing Statement

There will be no additional unpublished data available for this study.

Funding Statement

This work was supported by National Institute for Health Research grant number PB-PG-0215-36147.

Competing Interest Statement

There are no competing interests.

For peer review only - http://bmjopen.bmj.com/site/about/guidelines.xhtml

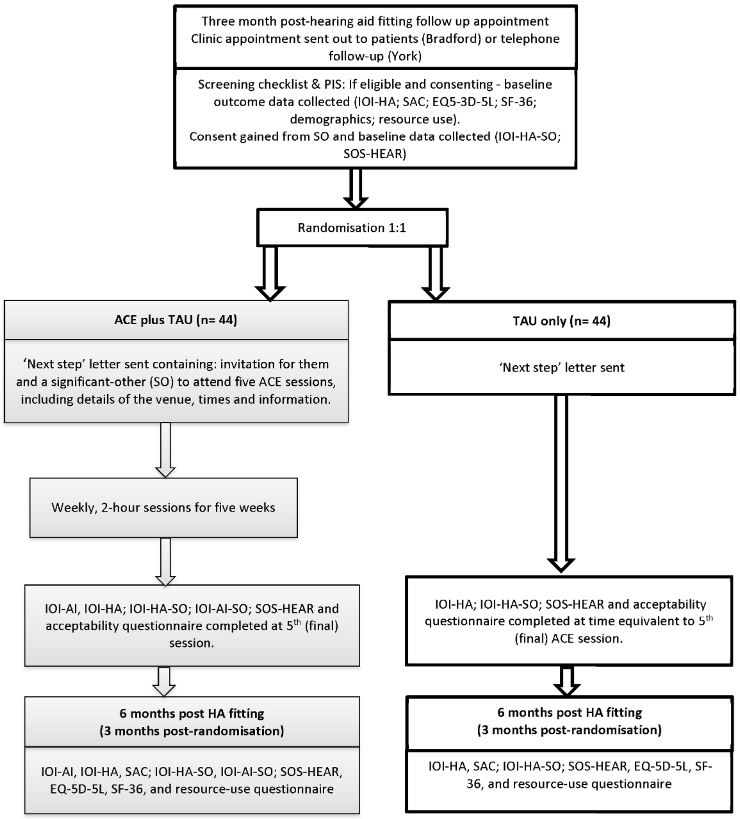

209x297mm (300 x 300 DPI)

BMJ Open

A RANDOMISED CONTROLLED FEASIBILITY TRIAL OF THE ACTIVE COMMUNICATION EDUCATION PROGRAMME PLUS HEARING AID PROVISION VERSUS HEARING AID PROVISION ALONE (ACE TO HEAR): A STUDY PROTOCOL.

Journal:	*BMJ Open*
Manuscript ID	bmjopen-2018-021502.R1
Article Type:	Protocol
Date Submitted by the Author:	23-Mar-2018

Complete List of Authors:	Thyer, Nicholas; University of Leeds, Leeds Institute of Cardiovascular and Metabolic Medicine (LICAMM)<br>Watson, Jude; University of York, Department of Health Sciences<br>Jackson, Cath; Valid Research<br>Hickson, Louise; The University of Queensland, Department of Speech and Hearing<br>Maynard, Christina; University of Leeds, Leeds Institute of Cardiovascular and Metabolic Medicine (LICAMM)<br>Forster, Anne; University of Leeds, Academic Unit of Elderly Care and Rehabilitat<br>CLARK, LAURA; University of York, Department of Health Sciences<br>Bell, Kerry; University of York, Health Sciences<br>Fairhurst, Caroline; University of York, Health Sciences<br>Cocks, Kim; University of York, Department of Health Sciences<br>Gardner, Rob; Bradford Teaching Hospitals NHS Foundation Trust<br>Iley, Kate; York Teaching Hospital NHS Foundation Trust, Audiology<br>Gailey, Lorraine; Hearing Link

**Primary Subject Heading**:	Health services research
Secondary Subject Heading:	Communication, Ear, nose and throat/otolaryngology
Keywords:	Hearing Loss, Active Communication Education, Hearing Aid Benefit, Economic Benefit, Communication Benefit, Hearing Aid Use

SCHOLARONE™
Manuscripts

For peer review only - http://bmjopen.bmj.com/site/about/guidelines.xhtml

A RANDOMISED CONTROLLED FEASIBILITY TRIAL OF THE ACTIVE COMMUNICATION EDUCATION PROGRAMME PLUS HEARING AID PROVISION VERSUS HEARING AID PROVISION ALONE (ACE TO HEAR): A STUDY PROTOCOL.

N J Thyer,[1] J Watson,[2] C Jackson,[3] L Hickson,[4] C Maynard, [1] A Forster, [5] L Clark,[2] K Bell,[2] C Fairhurst,[2] K Cocks,[2] R Gardner[6], K Iley, [7] L Gailey. [8]

Acknowledgments: The Authors would like to thank Catherine Hewitt[2] for helpful statistical advice and comments on an earlier draft of this paper and members of the Project Advisory Panel (PAP), Emmanuelle Blondiaux-Ding, Philip Le Mare and Shona Hudson for their invaluable advice on many aspects of the project.

Corresponding author:

Dr N J Thyer: n.j.thyer@leeds.ac.uk

Author affiliations:

[1]Leeds Institute of Cardiovascular and Metabolic Medicine (LICAMM,) University of Leeds, Leeds, UK

[2]York Trials Unit, Department of Health Sciences, University of York, ARRC Building, York, UK

[3]Valid Research Ltd, Wetherby, UK

[4]School of Health and Rehabilitation Sciences, The University of Queensland, Brisbane, Queensland 4072, Australia

[5]Leeds Institute of Health Sciences (LIHS), University of Leeds, Leeds, UK

[6]Audiology Department, Bradford Royal Infirmary, Bradford, UK

[7]Audiology Department, York Hospital, York, UK

For peer review only - http://bmjopen.bmj.com/site/about/guidelines.xhtml

[8]Hearing Link, Eastbourne, UK

For peer review only

For peer review only - http://bmjopen.bmj.com/site/about/guidelines.xhtml

Abstract:

Introduction: Up-to 30% of hearing aids fitted to new adult clients are reported to be of low benefit and used intermittently or not at all. Evidence suggests that additional interventions paired with service-delivery redesign may help improve hearing aid use and benefit. The range of interventions available is limited. In particular, the efficacy of interventions like the Active Communication Education (ACE) programme that focus on improving communication success with hearing impaired people and significant others, has not previously been assessed. We propose that improved communication outcomes associated with the ACE intervention, lead to an increased perception of hearing aid value and more realistic expectations associated with hearing aid use and ownership, which are reported to be key barriers and facilitators for successful hearing aid use. This study will assess the feasibility of delivering ACE and undertaking a definitive randomised controlled trial (RCT) to evaluate whether ACE would be a cost effective and acceptable way of increasing quality-of-life through improving communication and hearing aid use in a public health service such as the NHS.

Methods and analysis: This will be a randomised controlled, open feasibility trial with embedded economic and process evaluations delivered in audiology departments in two UK cities. We aim to recruit 84 patients (and up to 84 significant others) aged 18 years and over, who report moderate or less than moderate benefit from their new hearing aid. The feasibility of a larger scale study and the acceptability of the ACE intervention will be measured by recruitment rates, treatment retention, follow-up rates and qualitative interviews.

Ethics and dissemination: Ethical approval granted by South East Coast-Surrey Research Ethics Committee (16/LO/2012). Dissemination of results will be via peer reviewed research publications both online and in print, conference presentations, posters, patient forums and Trust bulletins.

Trial registration number: ISRCTN28090877

For peer review only

For peer review only - http://bmjopen.bmj.com/site/about/guidelines.xhtml

Strengths and limitations of this study:

- This will be the first study to evaluate the processes involved in delivering the ACE intervention in a GP referral pathway for new NHS hearing aid users.
- If the RCT is shown to be feasible then the data from this study will provide critical information that will inform the design of a larger RCT to determine the social, clinical and economic outcomes of the ACE in this important clinical pathway.
- The study is powered to allow the standard deviation of the proposed outcome measures to be estimated with reasonable certainty to inform future sample size calculations.
- This study is limited to assessing the feasibility of RCT and ACE delivery processes. Ultimately the test of whether the ACE intervention leads to longer term communication success, better hearing aid use, hearing aid benefit, quality-of-life and economic gains, will be tested in a future full-scale RCT.

For peer review only

For peer review only - http://bmjopen.bmj.com/site/about/guidelines.xhtml

INTRODUCTION

Age-related hearing impairment is a major world-wide public health issue for ageing populations [1]. It is reported as the third most common chronic condition affecting approximately 328 million (91%) middle and older aged adults[2], over 10 million adults in the UK alone.[3 4] By the age of 70, 70% will have a mild or worse hearing-impairment, progressively worsening with age.[5] Hearing impairment is commonly associated with reduced quality-of-life and well-being[6-8] including depression[9] and anxiety,[10] social isolation,[8] poor social interactions,[11-13] cognitive dysfunction,[14] increased risk of developing dementia and reduced emotional, behavioural and general social wellbeing.[15] In addition, disability in these domains is often experienced by normally-hearing significant others (SOs) living with hearing-impaired people.[16-18] Hearing impairment therefore represents an enormous burden on society and the economy.

In developed countries the most common treatment is to fit a hearing aid.[6] Despite strong evidence that hearing aid use is associated with reductions in hearing disability noted above [6 19-21] hearing aid use is remarkably low. [22 23] It is estimated that up to 30% of UK adult hearing aid owners do not use them regularly or at all.[4 24-26] International studies support these data.[8 27-29] Cost implications for the NHS are significant as they provide 80% of UK hearing aids,[25] fitting more than 300,000 new devices each year of which an estimated 20,000-120,000 are un-used. Even with global advances in technology, fitting protocols and outcome measurements,[30] there is little evidence that NHS hearing aid use and the expected gains in benefit and quality of life have improved over the last decade[19 25] and treatment continues to focus primarily on technology.

A recent systematic review of additional treatment found very low quality evidence that self-management and service delivery interventions may be of benefit in auditory rehabilitation.[31] However, the authors found no studies that examined the effect of these sorts of interventions on hearing aid use.

Reasons for hearing aid non-use are complex.[32] Research has identified psycho-social factors important for successful aural rehabilitation including; personal and societal attitudes to hearing-impairment[33] patient

For peer review only - http://bmjopen.bmj.com/site/about/guidelines.xhtml

involvement in decision making[34-37] and expectations of benefit and communication success in a range of communication situations.[38 39] Key barriers and facilitators to successful hearing aid use have been identified as being related to expectations of benefit and meaningful participation in everyday life.[40 41] The World Health Organisation's International Classification of Functioning, Disability and Health (ICF)[42] provides a functional description of difficulties (related to these expectations) experienced by hearing-impaired people and their hearing communication partners, for example, avoidance of difficult listening situations that lead to 'activity limitations' and 'participation restrictions'.

One intervention that is designed to reduce these limitations and restrictions is The Active Communication Education (ACE) programme (the focus of this paper). The ACE trains participants to develop solutions to specific difficult communication scenarios that commonly lead to their avoidance of or reduced participation in important activities. The effectiveness of ACE as an *alternative* intervention to a hearing aid has been evaluated and two small studies demonstrate ACE benefits in improving communication function and hearing related quality-of-life.[43 44] ACE effectiveness as an *adjunct* to hearing aid fitting has not been evaluated, although there is some weak evidence that supports its use in this context [44] i.e. longer-term improvements in using communication strategies being associated with hearing aid users.

A systematic review of group communication programme effectiveness conducted in 2005,[45] revealed just nine small and methodologically poor studies. The review reported weak evidence for short-term benefits related to reduced hearing disability; improvements in quality-of-life; hearing aid use and communication function[46] when interventions were delivered in concert with a hearing aid. The authors concluded that there was a clear need for large sufficiently powered randomized controlled studies to determine short and long term benefits of adult communication rehabilitation group interventions as an adjunct to hearing aid fitting. Such a study has yet to be completed. Evidence that post-dates this 2005 review does little to change the situation providing only weak additional evidence for moderate gains in hearing-loss related quality-of-life for communication-based group interventions.[47] There are indications that group

For peer review only

For peer review only - http://bmjopen.bmj.com/site/about/guidelines.xhtml

rehabilitation programmes like ACE have the potential to realise economic gains for service providers. For example, Abrams[48] estimated that a hearing aid plus a four-week group rehabilitation programme reduced the treatment cost per quality-adjusted life-year (QALY) gained by more than half; the cost of implementing the rehabilitation programme was less than 6% of the total rehabilitation cost per patient. Even so, with no strong evidence that such interventions delivered as an adjunct to hearing aid fitting are clinically worthwhile, they are not routinely offered in public or private hearing healthcare sectors in the UK.

In summary, there is some low quality evidence that ACE and similar programmes improve communication function and quality-of-life and these outcomes may be enhanced when delivered in conjunction with a hearing aid. Communication programmes involve a substantial commitment on the part of participants and those who run and pay for them. Recent evidence shows that it is effective and feasible to deliver ACE as an alternative intervention in Australia and Sweden.[43 44 49 50] There is now a need to establish whether reported clinical and economic benefits of ACE and communication programmes like ACE can be achieved in the context of NHS hearing aid provision. This protocol for The ACE To HEAR study (**A**ctive **C**ommunication **E**ducation **To** improve **HEAR**ing) is intended to deliver ACE to unsuccessful or struggling new NHS hearing aid users, three-months post-fitting, in order to assess whether a large RCT designed to evaluate the effectiveness of ACE in improving hearing aid benefit within the UK National Health Service (NHS) is feasible.

METHODS AND ANALYSIS

This protocol was developed and is reported according to the SPIRIT (Standard Protocol Items for Randomized Trials) statement.[51]

Study Aim:

The aim of this study is to determine the feasibility of delivering a future, full-scale randomised controlled trial (RCT) to evaluate ACE plus treatment-as-usual versus treatment-as-usual alone, within the UK National

For peer review only - http://bmjopen.bmj.com/site/about/guidelines.xhtml

Health Service (NHS) in two UK cities. Treatment-as-usual is defined as a referral from a patient's GP to audiology services to treat permanent hearing loss. It comprises up to two appointments for hearing aid fitting and a third face to face or telephone follow-up appointment.

Study objectives

Objectives will evaluate ACE delivery and trial delivery processes.

ACE Delivery Objectives

1) To assess ACE up-take rates, eligibility and acceptability of clinic location (between and within the two study sites) for participants and SOs.
2) To evaluate the level of ACE attendance and retention amongst participants randomised to the ACE arm of the study.
3) To assess acceptability of ACE with participants, SOs and audiologists.
4) To assess capability, capacity and willingness of audiology departments to support delivery of ACE within existing services.
5) To assess intervention fidelity of delivering ACE.

Trial Delivery Objectives

6) To assess RCT recruitment rates, evaluate the randomisation process and time to accrue ACE groups.
7) To assess the acceptability of study processes to participants, SOs and audiologists (i.e. those related to recruitment, the feasibility of identifying struggling hearing aid users, randomisation process, data collection, measurement of ACE fidelity and acceptability)
8) To explore patient reported outcome measures (PROMs) and estimate likely standard deviations, including quality-of-life tools (EQ-5D-5L; SF-36) and a bespoke health care resource use/acceptability/utility questionnaire for use in an intended full-scale RCT.

Study design

For peer review only - http://bmjopen.bmj.com/site/about/guidelines.xhtml

This study commenced on the 1st February 2017 and is of 24 months duration. The study design is a randomised controlled, open feasibility trial with embedded economic and process evaluations delivered in one audiology department in each of two UK cities. The design of the trial is shown in Figure 1.

Study setting

Study sites for this feasibility study are the Audiology Departments at York Hospital (YH), York Teaching Hospitals NHS foundation Trust, and the Bradford Royal Infirmary (BRI), Bradford Teaching Hospitals NHS Foundation Trust.

Study population

The study population will consist of adult patients aged 18 years or over, receiving treatment-as-usual delivered in one of the two participating centres. They will be considered potentially eligible if all of the following eligibility criteria apply at their 3-month post-fitting follow-up appointment:

Inclusion criteria:

a) Moderate or less than moderate benefit, defined by IOI-HA question 2.[52 53]
b) Hearing impairment: pure-tone average better ear thresholds at 500, 1000, 2000, and 4000 Hz of more than 25 dB HTL.
c) No significant self-reported history of neurological impairment.
d) Willing to provide written informed consent.
e) Able to provide written informed consent.
f) Able to take part in the intervention by understanding and using spoken English.
g) Able to self-complete the English language outcome measure tools.
h) The following inclusion criteria for Significant Others (SOs) will be assessed:
    a. A spouse or other family member who lives with or is a carer for a patient recruited to the study.

Exclusion criteria:

For peer review only

For peer review only - http://bmjopen.bmj.com/site/about/guidelines.xhtml

a) Severe or profound bilateral hearing impairment. Pure-tone better ear average thresholds measured at 500, 1000, 2000, and 4000 Hz of more than 85 dB HTL, since experience [43] suggests this group of patients may struggle to effectively participate in the intervention setting.
b) Significant on-going ear related health or mental health Issues that, in the audiologist's or associate audiologist's professional opinion, would preclude hearing aid fitting or attendance at ACE sessions.
c) Unable or unwilling to give written informed consent.

In addition, SOs will be excluded if they are unable or unwilling to give written informed consent.

Patients who do not have a SO or family member able to attend the ACE sessions are still eligible to participate in the study.

Sample size calculation and recruitment

As this is a feasibility study, the main purpose is to assess the acceptability and feasibility of conducting this study, with a view to designing and conducting a future full-scale trial.[54] Six ACE groups are planned with up to seven patients in each (five minimum). This leads to a maximum sample size of 44 patients and up to 44 significant others for the ACE intervention arm, and 44 in the control arm (n=88). This sample size will allow the standard deviation of the proposed outcome measures to be estimated with reasonable certainty to inform future sample size calculations.[54] Collectively, the two study sites fit approximately 4300 hearing aids a year and based on their experience we estimate 10% will require extra help at follow-up and be eligible for inclusion. Recruitment of patients commenced on the 1st April 2017 and is now underway. The recruitment window is currently planned to end on 28th February 2018 and there is potential to extend this phase until 30th April 2018 if necessary.

Randomisation

Eligible, consenting patients from the same study site who have completed baseline assessments will be randomised by a remote, centralised randomisation service (provided by York Trials Unit) in batches of 10-14 (intervention: control ratio of 1:1) using block randomisation in a single large block per batch. Following

For peer review only - http://bmjopen.bmj.com/site/about/guidelines.xhtml

randomisation, a letter outlining the next steps will be sent to participants. For those allocated to the ACE arm, this will contain an invitation for them and their SO to attend five ACE sessions, including details of the venue, times and information.

Blinding

By the nature of the interventions used within this study, blinding of the participants and facilitator is not possible. The research team responsible for data analysis and reporting will be blinded where possible.

Intervention allocation

Consenting participants will be randomly allocated to either:

a) **Treatment-as-usual:** Participants randomised to receive Treatment-as-usual will continue to receive usual care delivered by their service.

b) **ACE plus Treatment-as-usual**: Participants randomised to receive the Active Communication Education (ACE) plus Treatment-as-usual, will attend five two hour weekly sessions of the ACE programme, developed in Australia [43] and will continue to receive usual care delivered by their service.

The ACE programme will be delivered as described in the published ACE manual[55] by a trained facilitator to groups of hard of hearing people and SOs where possible. The same audiologist facilitator will deliver ACE to all groups.

ACE consists of six modules based on everyday communication activities known to be problematic for hard of hearing adults: Module 1: Communication needs analysis; Module 2: Conversation in background noise; Module 3: Conversation around the house; Module 4: Communication with difficult speakers; Module 5: Listening to other signals; and Module 6: Listening to public-address systems.

Through the use of demonstrations, practical exercises, discussion and problem solving, participants and significant others will learn individual problem-solving skills which can be applied in a range of novel

For peer review only - http://bmjopen.bmj.com/site/about/guidelines.xhtml

situations and discuss the use of communication strategies, lip reading skills, clarification skills, and assistive technology.

Intervention fidelity

Assessment of the fidelity to the ACE training of the audiologist facilitator and the delivery of the ACE programme will follow guidance from the NIH Behaviour Change Consortium, [56] and conducted in a pragmatic approach mirroring the way fidelity of an educational programme, such as ACE, would be realistically assured in an NHS context. The trainers will reflect on the training sessions; fidelity of ACE delivery will be *facilitated* by supervision of the audiologist for the first session, with feedback and self-reflection used to address any issues; fidelity of ACE delivery will then be *assessed* via a self-monitoring form recording on a 1-4 scale (poor – good) of their adherence to the ACE manual for each module across all sessions.

Intervention Compliance

Measuring compliance is challenging in complex interventions such as this, where there are a number of interacting elements (such as the influences of the ACE facilitator, participants' significant other, a varied selection of ACE module topics worked on and each individuals' perceptions of their (or their SO's) disability and treatment benefit). The intervention to be delivered is defined in the ACE manual[55] and our understanding (measurement) of what is actually delivered will be informed by the fidelity measures above, weekly attendance logs and weekly session records. ACE participants and SOs will self-report goal setting for each module and the number of goals achieved during the programme, the number of completed exercises (homework) will be reported providing an additional measure of the level of engagement or compliance with the programme that is focused on the participant and their SO.

Concurrent treatments

For peer review only - http://bmjopen.bmj.com/site/about/guidelines.xhtml

Any additional treatments identified will be available to both arms when necessary (e.g. hearing aid repairs, replacement batteries, etc.). The bespoke resource use questionnaire (see below) will allow us to monitor additional treatment accessed during the study.

Outcome Measures

Figure one shows the main processes of the trial, their relationship with the outcome measures and their timing. Data will be collected at baseline (prior to randomisation); during all ACE sessions and after ACE session 5 for ACE participants; and at an equivalent time for Treatment-as-usual participants; and approximately 3 months post-randomisation (6 months post-hearing aid fitting).

The feasibility and the potential for a future large-scale study design (the study's aim) will be measured by recording and evaluating:

ACE Delivery Outcomes:

1. Attendance: Attendance rates of participants and SOs at ACE sessions will be measured and who attends ACE session with the participant will be recorded (objective 2).
2. Fit of ACE with existing variations in service delivery models: Comparison of attendance at ACE delivered at different study sites and their satellite clinics and the effect of using telephone or face-to-face follow-up appointments will be recorded (objectives 1 and 4).
3. Can ACE be delivered as intended in the ACE protocol? This will be assessed through: (i) time taken to train the ACE facilitator successfully; (ii) number of ACE goals achieved by participants; and (iii) facilitator's adherence to the ACE protocol (fidelity) (objective 5).
4. Acceptability: Bespoke acceptability questionnaire regarding study processes, designed to explore participant and SO thoughts regarding the study including; ACE session organisation, session content, being approached and informed about the study and completing the questionnaires. The questionnaire is administered to participants and SOs at final ACE session and at a comparable time for the questions for Treatment-as-usual arm (objectives 3 and 4).

For peer review only

For peer review only - http://bmjopen.bmj.com/site/about/guidelines.xhtml

RCT Delivery Outcomes:

5. Recruitment: Number of follow-up cases in GP referred pathway; number of follow-up cases in GP referred pathway struggling with their hearing aid; number of and reasons for exclusions; number of patients who decline to participate and reason for declining; number who miss ACE intervention window (i.e. unable to attend an ACE group within 1-3 weeks after randomisation); number given an appointment for an ACE group session; and number of consented participants who fail to attend ACE sessions (objectives 6 and 7).
6. Allocation: Time taken to recruit and logistics of recruiting an optimally sized and located ACE group; time ACE started after randomisation (ACE intervention window); (objective 6).
7. Patient Reported Outcome Measure Data: Completion of the outcome measures below at each time-point will be recorded as well as extent of missing data within each outcome measure (objective 8)

Patient Reported Outcome Measures:

- International Outcomes Inventory for Hearing Aids (IOI-HA): [52] a seven-item questionnaire designed to evaluate the effectiveness of hearing aid treatments. The domains covered are: daily use; benefit; residual activity limitations; satisfaction; residual participation restrictions; impact on others; and quality of life. Responses are assigned a value from 1 to 5 and values summed. Higher scores indicate a more favourable outcome.
- International Outcomes Inventory for Alternative Interventions (IOI-AI): [57] a version of the IOI designed for use for non-hearing aid based interventions such as ACE, covering the same domains as the IOI-HA.
- Self-Assessment of Communication (SAC):[58] designed to measure the effect of hearing loss and hearing aid outcomes. This ten-item instrument covers questions about communication problems using a Likert

For peer review only - http://bmjopen.bmj.com/site/about/guidelines.xhtml

scale ranging from 1 ("almost never") to 5 ("practically always"). A percentage score is calculated by multiplying the raw score by 2, subtracting 20, and multiplying by 1.25.

- EQ-5D-5L:[59 60] A standardised generic instrument for describing and valuing health in terms of five dimensions (Mobility, Self-Care, Usual Activities, Pain/Discomfort, and Anxiety/Depression) using five levels of severity. Overall health on the day is also rated by the respondent on a 0–100 vertical visual analogue scale (EQ-VAS).
- Short-Form 36 (SF-36):[61] a generic health measure with 36 items assessing eight health concepts: physical functioning; role limitations due to physical problems; general health perceptions; vitality; social functioning; role limitations due to emotional problems; general mental health; and health transition

The following will be completed by participants' SOs only:

- International Outcome Inventory for Alternative Interventions: version for Significant Others (IOI-AI-SO):[56] a version of the IOI designed for use with SOs and non-hearing aid based interventions covering the same seven domains as the IOI-HA.
- International Outcomes Inventory for Hearing Aids: version for Significant Others (IOI-HA-SO):[56] an extension of the IOI-HA for use with the SO covering the same seven domains as the IOI-HA.
- Significant Other Scale for Hearing Disability (SOS-HEAR):[16] a 27-item self-report tool, which assesses third-party disability in spouses of older people with hearing impairment. It measures the effects of hearing impairment on the SO in the following domains: Changes to communication; Communication burden; Relationship changes; Going out and socializing; Emotional reactions to adaptations; Concern for partner. It uses a 5-point response scale: 0= no problem, to 4 = a complete problem.

The feasibility of collecting postal questionnaire data at each time point will be evaluated. Table 1 shows the data collection schedule.

For peer review only - http://bmjopen.bmj.com/site/about/guidelines.xhtml

Table 1: Data collection schedule: data are collected approximately three months post hearing aid fitting (Baseline); at each ACE session 1-5 for the intervention arm and at a time equivalent to ACE week 5 for the Treatment-as-usual arm; and approximately six months post hearing aid fitting. ~ ACE arm only. * Significant Others only

Study period:	Recruitment	Allocation	Post allocation					
Time point:	Baseline	0	ACE week 1	ACE week 2	ACE week 3	ACE week 4	ACE week 5	6 month
**RECRUITMENT:**								
Eligibility	•							
Informed consent	•							
Optional qualitative study consent	•							
Allocation		•						
**ASSESSMENTS:**								
Demographics	•							
IOI-HA	•						•	•
SAC	•							•
EQ-5D-5L	•							•
SF36	•							•
Resource use	•							•
IOI-AI~							•	•
ACE Participant attendance~			•	•	•	•	•	
ACE SO attendance~			•	•	•	•	•	
IOI-AI-SO~*							•	•
IOI-HA-SO*	•						•	•
SOS-HEAR*	•						•	•
Acceptability questionnaire							•	
Qualitative interviews (Participant & SO)							•	
Qualitative interviews (Audiologists)								•

For peer review only

For peer review only - http://bmjopen.bmj.com/site/about/guidelines.xhtml

Screening and enrolment

Patients attending Audiology Clinics at York Hospital (YH) and Bradford Royal Infirmary (BRI) will be approached to take part. Treatment-as-usual provided at the post hearing aid fitting follow-up will be according to site and therefore the recruitment process will vary slightly at each site:

a) York: patients will be followed up via a telephone interview. Eligibility will be checked during telephone interview and from medical records. Details of eligible and interested patients will, with their permission, be passed onto a non-ACE researcher who will post out a PIS and conduct a telephone follow-up call a few days later to see if still interested. Contact information is provided in the PIS so that the patient has opportunity to ask questions regarding the study. If willing to participate, informed consent and baseline questionnaire will be completed by post. SOs of patients who are recruited at York will receive a SO-specific PIS, consent form and baseline questionnaire by post to be returned in a freepost envelope.

b) Bradford: patients will be offered a face-to-face follow-up appointment 3-months post hearing aid fitting. Eligibility will be checked at this appointment and from medical records. Details of eligible and interested patients will be passed onto a non-ACE researcher who will provide a patient information sheet (PIS) and discuss the study. The patient will have the opportunity to ask questions and if willing, provide informed consent and complete a baseline questionnaire. If further consideration is required, the patient will be contacted by telephone call a few days later to see if still interested. In addition, if an SO attends the appointment with a patient, they will be provided with a SO-specific PIS, consent form and baseline questionnaire. Otherwise the documentation will be given to the patient to pass on to their SO or posted out.

We will monitor the proportion of patients referred to the Treatment-as-usual pathway during the study recruitment who subsequently do not attend or are not contactable by telephone for their post HA fitting

For peer review only - http://bmjopen.bmj.com/site/about/guidelines.xhtml

follow-up in order to estimate how many referrals may potentially be lost to recruitment. We will liaise with audiologists to identify reasons for non-attendance where possible.

Data collection and management

All data for the participant outcome measures will be collected by self-completed questionnaires returned by post or in secure boxes within the audiology clinics. Participants and SOs who fail to return their postal questionnaires will receive one reminder letter. Participants may also be asked to complete a questionnaire over the telephone, or asked to provide any missing data if required. Data from these paper forms will then be entered into a master database for the trial using either optical scanning techniques or entered manually.

Participants may withdraw from all or any aspects of the study without influencing their future care or treatment. A brief update of how the study is progressing will be sent out in order to maintain participant engagement with the study.

All information collected during the course of the trial will be kept strictly confidential. Information will be held securely on paper and electronically at York Trials Unit. All trial data will be identified using a unique trial identification number. Analytical datasets will not contain any identifiable information. Data will be archived for a period of at least 10 years following the end of the study.

Statistical analysis

A single analysis will be conducted at the end of the trial using Stata v13 or later. Data summaries and analyses will inform the design of a full-scale RCT of the intervention. Baseline data will be summarised by trial arm, using descriptive statistics for continuous data (mean, standard deviation (SD), median, minimum, maximum, number missing) and counts and percentages for categorical data. Recruitment rates will be reported monthly and overall, and by site. The flow of participants through the trial will be detailed in a CONSORT flow diagram and referral, consent and attendance rates will be summarised overall and by site using counts, percentages and 95% confidence intervals.

For peer review only

For peer review only - http://bmjopen.bmj.com/site/about/guidelines.xhtml

The number of ACE sessions attended will be summarised alongside any SOs who attended the sessions. Summaries will be provided overall, by site/clinic and by whether follow-up appointments were made as single or block booking. Acceptability data using Likert scales at 6 months, for participants, SOs and audiologists will be summarised separately using summary statistics and presented graphically using bar charts, by trial arm.

The number of participants withdrawing from the ACE intervention and/or the trial and any reasons for withdrawal will be summarised.

The time taken to train audiologists to deliver ACE and the number of ACE goals achieved by participants will be summarised. Fidelity scores (from self-monitoring forms) will be summarised overall and by session. The proportion of training and ACE intervention delivered as intended, as well as any adaptations to training/intervention will be reported. Variations in dose of ACE intervention will be measured through ACE attendance and attrition data.

Questionnaire return rates at each time point will be presented overall and by trial arm. PROMs at each time point will be summarised descriptively overall, by trial arm, and by ACE group for participants allocated to receive the ACE intervention. Standard deviations will be presented with 80% confidence intervals to inform future sample size calculations. The proportion of participants at the floor and ceiling of each measure, at each time point, will be reported along with the standardised response mean (SRM) to measure the sensitivity of each questionnaire to detect change. The SRM will be calculated as mean change in scores or values divided by the standard deviation in change scores.[62] Questionnaire completion times (from self-report) will be summarised as a consideration for instruments going into the full-scale evaluation. Missing data will be reported as a proportion of the total expected data set for each measure and will inform feasibility.

Qualitative data

Following delivery of all the ACE intervention sessions:

For peer review only - http://bmjopen.bmj.com/site/about/guidelines.xhtml

- The facilitator will be interviewed to explore the training and implementation process and their experiences of delivering ACE including barriers/facilitators to adhering to the ACE protocol.
- Up to three audiologists from both study sites (up to six in total) will also be interviewed, exploring the capability, capacity and willingness of their audiology departments to support the ACE study within their existing services. The acceptability of study processes will also be explored.
- A sample of 10-12 participants in the ACE intervention arm and four participants in the control arm (with their SOs if willing) will also be interviewed as soon as possible after the completion of the ACE sessions. We will purposively select participants to ensure a mix of those with good/poor hearing aid outcomes (measured at the 5th and final ACE session and equivalent control arm time point, see Fig 1. for outcome measures) as well as ensuring we include some participants who dropped out of the sessions/study, ensuring a wide range of views are collected. Control arm participants will include those who have dropped out from the study where possible, allowing us to explore reasons for this. SOs will be interviewed as part of a dyad with the participant. Semi-structured interviews will explore the acceptability of the ACE (e.g. venue, timing, content), its perceived impact (reflecting on hearing aid outcomes) with ACE intervention arm participants; and views on study processes (e.g. recruitment, outcome measures and timing) with ACE intervention and control arm participants.

Interviews will be audio-recorded, transcribed verbatim and analysed (with NVivo-11) by the research team led by CJ, using the Framework approach[63] which is particularly useful for analysing qualitative data in a pragmatic yet systematic way, where theoretical development is not needed. The steps are familiarisation, construction of a thematic framework, indexing and charting the data, mapping and interpretation.

Economic analysis and quality of life data

A full cost-effectiveness analysis will not be conducted as this is a feasibility trial thus the study is not powered to detect significant differences.

For peer review only - http://bmjopen.bmj.com/site/about/guidelines.xhtml

The costs of implementing the ACE intervention will be estimated and the potential resource implications versus usual care will be explored. Resource use will be summarised by resource use type (e.g. GP appointments, outpatient appointments) and appropriate unit costs to be applied to each resource use type will be identified. These will be sourced from a combination of local costings and national databases.[64] The costing approach will take a broad analytical perspective accounting not only for NHS costs but also for those observed by patients, though this cost will be presented separately. It is anticipated that additional resources utilised in the ACE intervention arm will largely be NHS staff time and travel/time for patients and SOs, patients and SOs use of primary and secondary NHS care, any private treatments attended, whether related to their hearing or for any other reason, changes to medication and employment or recreational activities. A draft resource use questionnaire based on these anticipated additional resources was designed for this study. The questionnaire will be developed further and tested during the feasibility trial to ensure that all relevant and necessary data can be collected to establish a reliable and valid tool with which to capture resource use for a future full economic evaluation.

Methods to estimate an incremental cost-effectiveness ratio for the ACE intervention versus Treatment-as-usual alone in terms of Quality Adjusted Life Years (QALYs) will be explored. No health-related quality of life assessment tool is currently sufficiently sensitive to all populations, and in the field of hearing health, there has been limited research to identify the most effective tool. In the UK, NICE advocates the used of the EQ-5D for generating quality adjusted life years, though it is acknowledged that this is not always the most sensitive tool for particular populations for whom the majority of its dimensions may not apply.[65] In the US analysis of a four week rehabilitation programme noted above,[48] the SF-36 was used to generate QALYs rather than the EQ-5D, with the SF-36 showing a reduction in the cost per QALY in favour of the intervention. For the present feasibility trial, both assessments will be used to elicit QALYs and a comparison will be made between the outcomes of the two measures. This will enable a decision to be made as to which tool would be most appropriate in the full-scale trial.

For peer review only - http://bmjopen.bmj.com/site/about/guidelines.xhtml

The feasibility work will also be used to help to identify any patterns of missing data and any issues relevant for sensitivity analysis which will influence statistical plans for dealing with imprecision and other uncertainties in the full RCT. For example, data can be bootstrapped to account for the expected skewness evident in economic cost data. The data collected as part of this feasibility study will be used to inform subsequent pre-trial modelling.

Adverse events

Risks within this study are considered to be minimal. It is considered highly unlikely that the ACE intervention arm will suffer any adverse consequences as a result of receiving the ACE plus Treatment-as-usual. Nevertheless, interviews with ACE participants, the ACE facilitator and ACE questionnaire data will be used to monitor this eventuality.

Trial monitoring and oversight

Due to the low risk nature of this trial, approval has been obtained to set up one independent steering and monitoring committee to undertake the roles traditionally undertaken by the Trial Steering Committee and Data Monitoring and Ethics Committee. Regular meetings of a Study Management Group will take place to oversee the progress of the study and review recruitment. We will establish a Project Advisory Panel (PAP) with between two and four hard of hearing adults or hearing spouses that will meet a minimum of five times over the duration of the project. The PAP is a group of patients, service users, carers and lay members of the public whose role is to support and advise the Study Management Group on all aspects of the study's progression and management.

Patient and Public Involvement

Three PPI activities informed the development of this application. First, a funded public engagement event about public perceptions of hearing-impairment was held at the Thackeray Medical Museum in Leeds. This event helped to inform the research question. Participants identified a need for wider availability of

For peer review only - http://bmjopen.bmj.com/site/about/guidelines.xhtml

treatments additional to hearing-aids and that non-technological interventions for hearing-impaired people were a priority. Delegates identified communication education as a useful addition to hearing-aid use for many hearing-impaired people and their family members. This feedback informed the study design in the following way: the choice of an interactive communication based intervention rather than an informational one; the need to ensure that routine practical information about hearing-aids and hearing-impairment is delivered consistently and checked after fitting.

Second, a focus group was held to consult with service-users on the proposed research question, study design and intervention delivery. Four participants were asked to discuss (a) study information and consent procedures, (b) factors that might encourage or discourage their participation in the proposed study such as the burden of the intervention, (c) types of communication scenarios that are important to them, (d) factors that might motivate them to be more active communicators. The outcomes informed our recruitment strategy to maximise interest, commitment and recruitment rates. The group's views helped develop study information sheets and operational components of delivering ACE.

Third, the charity Hearing Link, who have extensive experience of PPI and managing and delivering group interventions of this type, were consulted about involving public and patients in operationalising and delivering ACE. Patients and service users and carers are involved in the conduct of this study as active members of the Project Advisory Panel (PAP).

We will present the findings of this study in patient forums, Trust bulletins and PPI activities including newsletters and public interest groups who work and support older adults with hearing impairment.

Data Monitoring and management

For peer review only - http://bmjopen.bmj.com/site/about/guidelines.xhtml

Information relating to study participants will be kept confidential and managed in accordance with the Data Protection Act, NHS Caldicott Guardian, Research Governance Framework for Health and Social Care and the Research Ethics Committee (REC) approval.

Participant details will be stored on a secure password protected server located at the University of York, for the purposes of assisting in follow-ups during the study. All paper data collected from participants will be maintained in a safe secure environment at York Trials Unit. Paper records will be identified using identifiers rather than personally identifiable information. Analytical datasets will not contain any identifiable information.

The confidentiality of the participants, SOs and audiologists interviewed during their qualitative interviews will be ensured by assigning a unique identification code to electronic sound files and transcripts of interviews, known only to the qualitative researcher and appropriate members of the research team. Any quotes published will be anonymous further protecting participant confidentiality.

ETHICS AND DISSEMINATION

Ethical approval has been granted by South East Coast—Surrey Research Ethics Committee (16/LO/2012).

Since the study started in February 2017, three HRA approved amendments were added to the protocol and are included in the final version reported here:

1. Revised the fidelity check tool. This was considered a non-substantial amendment.
2. Remove inclusion criteria of less than three hours hearing aid use a day and adjusted inclusion criteria b) to include moderate benefit. We also gained approval to distribute study information flyers to study site staff and patients. These were considered substantial amendments.

For peer review only - http://bmjopen.bmj.com/site/about/guidelines.xhtml

3. Developed a study information flyer to be sent to patients not contactable by telephone for follow-up at the York study site. This was considered a non-substantial amendment.

The proposed study will be conducted in accordance with the MRC Guidelines on Good Clinical Practice in Clinical Trials.

The results from this study will be submitted to the funders, peer-reviewed journals, presented at relevant meetings/conferences and within the participating and other audiology departments. We also intend to present the findings of this study in patient forums, Trust bulletins and PPI activities including newsletters and public interest groups who work and support older adults with hearing impairment.

CONCLUSION

This will be the first RCT of this type of group communication programme in the context of a public health service and as an adjunct to hearing aid fitting. The impact of this study will ultimately be realised by a larger fully powered RCT designed to determine the effectiveness of the ACE intervention in improving hearing aid benefit for hearing aid users within the NHS GP referral for a hearing aid pathway in the UK. The outcomes of this study will inform such a RCT.

The feasibility study will be deemed successful if:

1. 70% of recruitment targets attained for all research components.
2. Study consent/retention rates and proposed sample sizes, indicate delivery of the full RCT is plausible within a 5 year study period.
3. 90% of ACE groups of 5-7 consented participants formed within the intervention window with participants attending 3 of 5 sessions.
4. Economic, acceptability, outcome measure, and fidelity evaluation data successfully collected.
5. Participants, significant-others and audiologists evaluate acceptability of the ACE and RCT positively.

(Measures with over 10% missing data maybe modified/replaced prior to the main trial)

The criteria for success will result in one of following outcomes:

1. stop: full-scale RCT not be feasible in NHS setting
2. continue: feasible with modifications

For peer review only - http://bmjopen.bmj.com/site/about/guidelines.xhtml

3. continue: feasible with no modifications, close monitoring
4. continue feasible as is

For peer review only

For peer review only - http://bmjopen.bmj.com/site/about/guidelines.xhtml

FIGURE CAPTIONS

Figure 1: ACE To HEAR Study flow diagram. The diagram was developed using CONSORT guidelines (http://www.consort-statement.org/) and indicates the main processes of the trial, their relationship with the outcome measures and their timing.

For peer review only

For peer review only - http://bmjopen.bmj.com/site/about/guidelines.xhtml

REFERENCES

1. Organisation WH. WHO global estimates on prevalence of hearing loss: Mortality and Burden of Diseases and Prevention of Blindness and Deafness. Secondary WHO global estimates on prevalence of hearing loss: Mortality and Burden of Diseases and Prevention of Blindness and Deafness. 2012. http://www.who.int/pbd/deafness/WHO_GE_HL.pdf.
2. Collins JG. Prevalence of selected chronic conditions: United States 1990-1992. Vital Health Stat 10(194) 1997.
3. Davis AC. The Prevalence of Hearing Impairmrent and Reported Hearing Disability Among Adults in Great Britain. International Journal of Epidemiology 1989;**18**(4):911-17.
4. AoHL. Hearing Matters: Taking action on hearing loss in the 21st century. http://www.actiononhearingloss.org.uk/supporting-you/policy-research-and-influencing/research/hearing-matters.aspx: Action on Hearing Loss, 2011.
5. Roth TN, Hanebuth D, Probst R. Prevalence of age-related hearing loss in Europe: a review. European Archives of Oto-Rhino-Laryngology 2011;**268**(8):1101-07.
6. Bess FH, Lichtenstein MJ, Logan SA, et al. Hearing impairment as a determinant of function in the elderly. J Am Geriatr Soc 1989;**37**(11):123-28.
7. Chia EM, Wang JJ, Rochtchina E, et al. Hearing impairment and health-related quality of life: The blue mountains hearing study. Ear and Hearing 2007;**28**:187-95.
8. Health Quality Ontario. Social isolation in community-dwelling seniors: an evidence-based analysis. Ontario health technology assessment series 2008;**8**(5):1-49.
9. Pronk, M., Deeg, D.J.H., Smits, C., et al. Prospective effects of hearing status on loneliness and depression in older persons: Identification of subgroups. International Journal of Audiology. 2011, **50**(12), pp.887-896.

For peer review only - http://bmjopen.bmj.com/site/about/guidelines.xhtml

10. Gopinath B, Schneider J, Hickson L, et al. Hearing handicap, rather than measured hearing impairment, predicts poorer quality of life over 10 years in older adults. Maturitas 2012;**72**(2):146-51.

11. Hetu R, Jones L, Getty L. The impact of acquired hearing impairment on intimate-relationships - implications for rehabilitation Audiology 1993;**32**(6):363-81.

12. Heine C, Browning C. The communication and psychosocial perceptions of older adults with sensory loss: a qualitative study. Ageing and Society 2004;**24**:113-30.

13. Heine C, Browning CJ. Communication and psychosocial consequences of sensory loss in older adults: overview and rehabilitation directions. Disability and Rehabilitation 2002;**24**(15):763-73.

14. Humes LE, Dubno JR, Gordon-Salant S, et al. Central Presbycusis: A Review and Evaluation of the Evidence. Journal of the American Academy of Audiology 2012;**23**(8):635-66.

15. Stephens D, Kramer SE. Living with hearing loss: The Process of Enablement. . London: Wiley, 2009.

16. Scarinci N, Worrall L, Hickson L. The effect of hearing impairment in older people on the spouse: Development and psychometric testing of The Significant Other Scale for Hearing Disability (SOS-HEAR). International Journal of Audiology 2009;**48**(10):671-83.

17. Wallhagen MI, Strawbridge WJ, Shema SJ, et al. Impact of self-assessed hearing loss on a spouse: A longitudinal analysis of couples. Journals of Gerontology Series B-Psychological Sciences and Social Sciences 2004;**59**(3):S190-S96.

18. Scarinci N, Worrall L, Hickson L. Factors Associated With Third-Party Disability in Spouses of Older People With Hearing Impairment. Ear and Hearing 2012;**33**(6):698-708.

19. Kochkin S. MarkeTrak VIII patients report improved quality of life with hearing aid usage. Hearing Journal 2011;**64**(6):25-32.

20. Laplante-Levesque A, Hickson L, Worrall L. Rehabilitation of Older Adults With Hearing Impairment: A Critical Review. Journal of Aging and Health 2010;**22**(2):143-53.

For peer review only

For peer review only - http://bmjopen.bmj.com/site/about/guidelines.xhtml

21. Ferguson MA, Kitterick PT, Chong LY, et al. Hearing aids for mild to moderate hearing loss in adults. Cochrane Database of Systematic Reviews 2017(9).

22. RNID. Royal National Institute for the Deaf. Information and resources. Secondary Royal National Institute for the Deaf. Information and resources 2011. http://www.rnid.org.uk/information_resources/

23. Davis A, Smith P, Ferguson M, et al. Acceptability, benefit and costs of early screening for hearing disability: a study of potential screening tests and models. Health Technology Assessment 2007;**11**(42).

24. Gianopoulos I, Stephens D, Davis A. Follow up of people fitted with hearing aids after adult hearing screening: the need for support after fitting. BMJ 2002;**325**.

25. Hougaard S, Ruf S. EuroTrak I: A consumer survey about hearing aids in Germany, France and the UK. First global comparative study of hearing, hearing loss, and hearing aids. The Hearing Review 2011;**18**(2):12-28.

26. Stephens D, Lewis P, Davis A, et al. Hearing aid possession in the population: Lessons from a small country. Audiology 2001;**40**(2):104-11.

27. Lupsakko TA, Kautiainen JH, Sulkava R. The non-use of hearing aids in people aged 75 years and over in the city of Kuopio in Finland. European Archives of Oto-Rhino-Laryngology 2005;**262**:165-69.

28. Popelka MM, Cruickshanks KJ, Wiley TL, et al. Low prevalence of hearing aid use among older adults with hearing loss: The epidemiology of hearing loss study. Journal of the American Geriatrics Society 1998;**46**(9):1075-78.

29. Smeeth L, Fletcher AE, Siu-Woon Ng E, et al. Reduced hearing, ownership, and use of hearing aids in elderly people in the UK-the MRC Trial of the Assessment and Management of Older People in the Community: a cross-sectional survey. The Lancet 2002;**359**(9316):1466-70.

30. Department of Health. Modernising NHS hearing aid services London: Department of Health, 2000.

31. Barker F, Mackenzie E, Elliott L, et al. Interventions to improve hearing aid use in adult auditory rehabilitation (Review). Cochrane Database of Systematic Reviews 2016; **Art. No.: CD010342**(8).

For peer review only - http://bmjopen.bmj.com/site/about/guidelines.xhtml

32. Ng JH-Y, Loke AY. Determinants of hearing aid adoption and use among the elderly: A systematic review. International Journal of Audiology 2015;**54**(5):291-300.

33. Wallhagen MI. The Stigma of Hearing Loss. Gerontologist 2010;**50**(1):66-75.

34. Laplante-Levesque A, Hickson L, Worrall L. What Makes Adults With Hearing Impairment Take Up Hearing Aids or Communication Programs and Achieve Successful Outcomes? Ear and Hearing 2012;**33**(1):79-93.

35. Laplante-Lévesque A, Hickson L, Worrall L. Factors influencing rehabilitation decisions of adults with acquired hearing impairment. International Journal of Audiology 2010;**49**(7):497-507.

36. Laplante-Levesque A, Knudsen LV, Preminger JE, et al. Hearing help-seeking and rehabilitation: Perspectives of adults with hearing impairment. International Journal of Audiology 2012;**51**(2):93-102.

37. Southall K, Gagne JP, Leroux T. Factors that influence the use of assistance technologies by older adults who have a hearing loss. International Journal of Audiology 2006;**45**(4):252-59.

38. Scarinci N, Worrall L, Hickson L. The effect of hearing impairment in older people on the spouse. International Journal of Audiology 2008;**47**(3):141-51.

39. Scarinci N, Worrall L, Hickson L. The ICF and third-party disability: Its application to spouses of older people with hearing impairment. Disability and Rehabilitation 2009;**31**(25):2088-100.

40. Gagne JP, Jennings MB, Southall K. The ICF: A Classification System and Conceptual Framework Ideal for Audiological Rehabilitation. Perspectves on Aural Rehabilitation and its Instrumentation 2009;**16**(1):8-14.

41. Lockey K, Jennings MB, Shaw L. Exploring hearing aid use in older women through narratives. International Journal of Audiology 2010;**49**(8):542-49.

42. WHO. ICF: International Classification of Fuctioning, Disability and Health. Geneva: World Health Organisation, 2001.

For peer review only - http://bmjopen.bmj.com/site/about/guidelines.xhtml

43. Hickson L, Worrall L, Scarinci N. A randomized control trial evaluating the Active Communication Education program for older people with hearing impairment. Ear and Hearing 2007;**28**(2):212-30.

44. Oberg M, Bohn T, Larsson U. Short- and Long-Term Effects of the Modified Swedish Version of the Active Communication Education (ACE) Program for Adults with Hearing Loss. Journal of the American Academy of Audiology 2014;**25**(9):848-58.

45. Hawkins DB. Effectiveness of counseling-based adult group aural rehabilitation programs: a systematic review of the evidence. Journal of the American Academy of Audiology 2005;**16**(7):485-93.

46. Chisolm TH, Abrams HB, McArdle R. Short- and long-term outcomes of adult audiological rehabilitation. Ear and Hearing 2004;**25**(5):464-77.

47. Preminger JE. Issues associated with the measurement of psychosocial benefits of group audiologic rehabilitation programs. Trends Amplif 2007;**11**(2):113-23.

48. Abrams H, Chisolm TH, McArdle R. A cost-utility analysis of adult group audiologic rehabilitation: Are the benefits worth the cost? Journal of Rehabilitation Research and Development 2002;**39**(5):549-57.

49. Hickson L, Worrall L. Beyond hearing aid fitting: improving communication for older adults. International Journal of Audiology 2003;**42**:S84-S91.

50. Oberg M, Bohn T, Larsson U, et al. A Preliminary Evaluation of the Active Communication Education Program in a Sample of 87-Year-Old Hearing Impaired Individuals. Journal of the American Academy of Audiology 2014;**25**(2):219-28.

51. Chan A, Tetzlaff JM, Altman DG, et al. Spirit 2013 statement: Defining standard protocol items for clinical trials. Annals of Internal Medicine 2013;**158**(3):200-07.

52. Cox RM, Alexander GC. The International Outcome Inventory for Hearing Aids (IOI-HA): psychometric properties of the English version. International Journal of Audiology 2002;**41**(1):30-35.

53. Cox RM, Alexander GC, Beyer CM. Norms for the international outcome inventory for hearing aids. Journal of the American Academy of Audiology 2003;**14**(8):403-13.

For peer review only

For peer review only - http://bmjopen.bmj.com/site/about/guidelines.xhtml

54. Teare M, Dimairo M, Shephard N, et al. Sample size requirements to estimate key design parameters from external pilot randomised controlled trials: a simulation study. Trials 2014;**15**(1):264.

55. Hickson L, Worrall L, Scarinci N, editors. *ACE: Active Communication Education.* : University of Queensland, 2015.

56. Bellg AJ, Resnick B, Minicucci DS, et al. Enhancing Treatment Fidelity in Health Behavior Change Studies: Best Practices and Recommendations From the NIH Behavior Change Consortium. Health Psychology 2004;**23**(5):441-51.

57. Noble W. Extending the IOI to significant others and to non-hearing aid-based interventions. International Journal of Audiology 2002;**41**(1):27-29.

58. Schow RL, Nerbonne MA. communication screening profile - use with elderly clients. Ear and Hearing 1982;**3**(3):135-47.

59. Herdman M, Gudex C, Lloyd A, et al. Development and preliminary testing of the new five-level version of EQ-5D (EQ-5D-5L). Quality of Life Research. Quality of Life Research 2011;**20**(10):1727-36.

60. Janssen M, Pickard A, Golicki D, et al. Measurement properties of the EQ-5D-5L compared to the EQ-5D-3L across eight patient groups: a multi-country study. Quality of Life Research 2013;**22**(7):1717-27.

61. Ware J, Sherbourne C. The MOS 36-item short-form health survey (SF-36) 1: conceptual framework and item selection. Med Care 1992;**30**:473-83.

62. Ritchie J, Lewis J. *Qualitative Research Practice*. London: Sage Publications, 2003.

63. Stratford PW, Binkley J, Solomon P, et al. Defining the minimum level of detectable change for the Roland-Morris questionnaire. Physical Therapy 1996;**76**(4):359-65.

64. Curtis L. Unit Costs of Health and Social Care 2014, 2014.

65. NICE. Guide to the Methods of Technology Appraisal.: National Institute of Health and Clinical Excellence: London, 2013.

For peer review only - http://bmjopen.bmj.com/site/about/guidelines.xhtml

Contributorship Statement

NJT led on the conception, design and writing of the study and study protocol with substantial contributions to the design, writing, critical review of intellectual content and final manuscript approval from JW, CJ, KC, AF, LH, LC, KB, CF, CM,RG, KI and LG. All authors agree to be accountable for their work. As Principle Investigator, NJT takes overall responsibility for the work.

KC provided statistical expertise in the study design and development stages of the project and the protocol. CF provided further essential statistical advice and expertise on the study protocol.

JW, CJ and LC made substantial contributions to the trial design and management. AF was involved in all aspects of the study and LG provided particular input to PPI. KB was specifically responsible for the health economic aspects of the study design and KI and RG were responsible for aspects specific to Trust's service delivery, providing expert clinical support.

Data Sharing Statement

There will be no additional unpublished data available for this study.

Funding Statement

This paper presents independent research funded by the National Institute for Health Research (NIHR) under its Research for Patient Benefit (RfPB) Programme (Grant Reference Number PB-PG-0215-36147). The views expressed are those of the author(s) and not necessarily those of the NHS, the NIHR or the Department of Health and Social Care..

Competing Interest Statement

There are no competing interests.

For peer review only

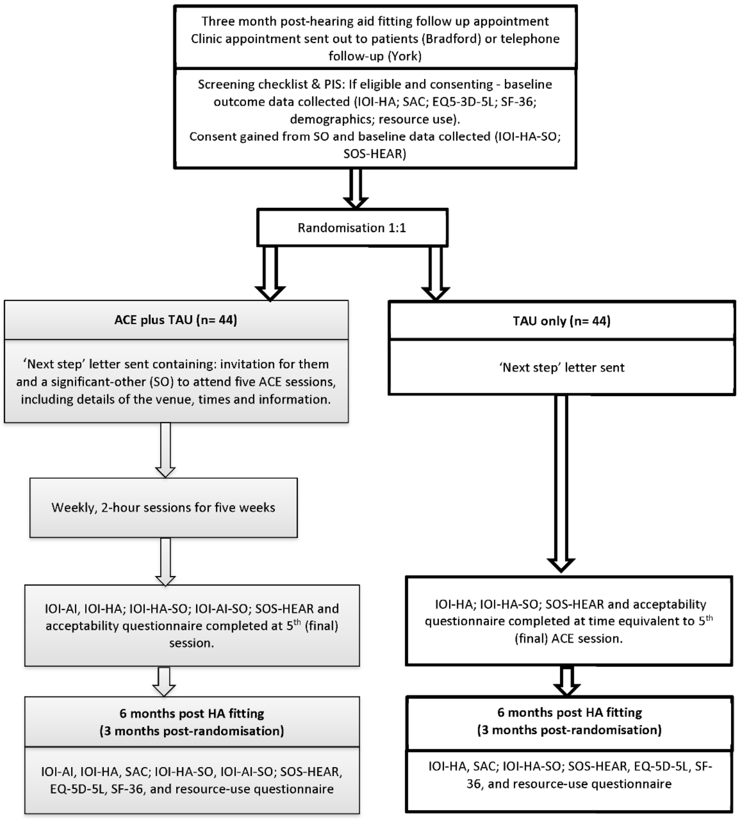

209x297mm (300 x 300 DPI)

BMJ Open

A RANDOMISED CONTROLLED FEASIBILITY TRIAL OF THE ACTIVE COMMUNICATION EDUCATION PROGRAMME PLUS HEARING AID PROVISION VERSUS HEARING AID PROVISION ALONE (ACE TO HEAR): A STUDY PROTOCOL.

Journal:	*BMJ Open*
Manuscript ID	bmjopen-2018-021502.R2
Article Type:	Protocol
Date Submitted by the Author:	23-May-2018
Complete List of Authors:	Thyer, Nicholas; University of Leeds, Leeds Institute of Cardiovascular and Metabolic Medicine (LICAMM)<br>Watson, Jude; University of York, Department of Health Sciences<br>Jackson, Cath; Valid Research<br>Hickson, Louise; The University of Queensland, Department of Speech and Hearing<br>Maynard, Christina; University of Leeds, Leeds Institute of Cardiovascular and Metabolic Medicine (LICAMM)<br>Forster, Anne; University of Leeds, Academic Unit of Elderly Care and Rehabilitat<br>CLARK, LAURA; University of York, Department of Health Sciences<br>Bell, Kerry; University of York, Health Sciences<br>Fairhurst, Caroline; University of York, Health Sciences<br>Cocks, Kim; University of York, Department of Health Sciences<br>Gardner, Rob; Bradford Teaching Hospitals NHS Foundation Trust<br>Iley, Kate; York Teaching Hospital NHS Foundation Trust, Audiology<br>Gailey, Lorraine; Hearing Link
**Primary Subject Heading**:	Health services research
Secondary Subject Heading:	Communication, Ear, nose and throat/otolaryngology
Keywords:	Hearing Loss, Active Communication Education, Hearing Aid Benefit, Economic Benefit, Communication Benefit, Hearing Aid Use

SCHOLARONE™
Manuscripts

A RANDOMISED CONTROLLED FEASIBILITY TRIAL OF THE ACTIVE COMMUNICATION EDUCATION PROGRAMME PLUS HEARING AID PROVISION VERSUS HEARING AID PROVISION ALONE (ACE TO HEAR): A STUDY PROTOCOL.

N J Thyer,[1] J Watson,[2] C Jackson,[3] L Hickson,[4] C Maynard, [1] A Forster, [5] L Clark,[2] K Bell,[2] C Fairhurst,[2] K Cocks,[2] R Gardner[6], K Iley, [7] L Gailey. [8]

Acknowledgments: The Authors would like to thank Catherine Hewitt[2] for helpful statistical advice and comments on an earlier draft of this paper and members of the Project Advisory Panel (PAP), Emmanuelle Blondiaux-Ding, Philip Le Mare and Shona Hudson for their invaluable advice on many aspects of the project.

Corresponding author:

Dr N J Thyer: n.j.thyer@leeds.ac.uk

Author affiliations:

[1]Leeds Institute of Cardiovascular and Metabolic Medicine (LICAMM,) University of Leeds, Leeds, UK

[2]York Trials Unit, Department of Health Sciences, University of York, ARRC Building, York, UK

[3]Valid Research Ltd, Wetherby, UK

[4]School of Health and Rehabilitation Sciences, The University of Queensland, Brisbane, Queensland 4072, Australia

[5]Leeds Institute of Health Sciences (LIHS), University of Leeds, Leeds, UK

[6]Audiology Department, Bradford Royal Infirmary, Bradford, UK

[7]Audiology Department, York Hospital, York, UK

[8]Hearing Link, Eastbourne, UK

Sponsor: Bradford Teaching Hospitals NHS Foundation Trust, Bradford Royal Infirmary, Bradford BD9 6RJ
Protocol version 3.0 17/10/2017

For peer review only

For peer review only - http://bmjopen.bmj.com/site/about/guidelines.xhtml

Abstract:

Introduction: Up-to 30% of hearing aids fitted to new adult clients are reported to be of low benefit and used intermittently or not at all. Evidence suggests that additional interventions paired with service-delivery redesign may help improve hearing aid use and benefit. The range of interventions available is limited. In particular, the efficacy of interventions like the Active Communication Education (ACE) programme that focus on improving communication success with hearing impaired people and significant others, has not previously been assessed. We propose that improved communication outcomes associated with the ACE intervention, lead to an increased perception of hearing aid value and more realistic expectations associated with hearing aid use and ownership, which are reported to be key barriers and facilitators for successful hearing aid use. This study will assess the feasibility of delivering ACE and undertaking a definitive randomised controlled trial (RCT) to evaluate whether ACE would be a cost effective and acceptable way of increasing quality-of-life through improving communication and hearing aid use in a public health service such as the NHS.

Methods and analysis: This will be a randomised controlled, open feasibility trial with embedded economic and process evaluations delivered in audiology departments in two UK cities. We aim to recruit 84 patients (and up to 84 significant others) aged 18 years and over, who report moderate or less than moderate benefit from their new hearing aid. The feasibility of a larger scale study and the acceptability of the ACE intervention will be measured by recruitment rates, treatment retention, follow-up rates and qualitative interviews.

Ethics and dissemination: Ethical approval granted by South East Coast-Surrey Research Ethics Committee (16/LO/2012). Dissemination of results will be via peer reviewed research publications both online and in print, conference presentations, posters, patient forums and Trust bulletins.

Trial registration number: ISRCTN28090877 https://www.isrctn.com/ISRCTN28090877

For peer review only - http://bmjopen.bmj.com/site/about/guidelines.xhtml

Strengths and limitations of this study:

- This will be the first study to evaluate the processes involved in delivering the ACE intervention in a GP referral pathway for new NHS hearing aid users.
- If the RCT is shown to be feasible then the data from this study will provide critical information that will inform the design of a larger RCT to determine the social, clinical and economic outcomes of the ACE in this important clinical pathway.
- The study is powered to allow the standard deviation of the proposed outcome measures to be estimated with reasonable certainty to inform future sample size calculations.
- This study is limited to assessing the feasibility of RCT and ACE delivery processes. Ultimately the test of whether the ACE intervention leads to longer term communication success, better hearing aid use, hearing aid benefit, quality-of-life and economic gains, will be tested in a future full-scale RCT.

For peer review only

For peer review only - http://bmjopen.bmj.com/site/about/guidelines.xhtml

INTRODUCTION

Age-related hearing impairment is a major world-wide public health issue for ageing populations [1]. It is reported as the third most common chronic condition affecting approximately 328 million (91%) middle and older aged adults[2], over 10 million adults in the UK alone.[3 4] By the age of 70, 70% will have a mild or worse hearing-impairment, progressively worsening with age.[5] Hearing impairment is commonly associated with reduced quality-of-life and well-being[6-8] including depression[9] and anxiety,[10] social isolation,[8] poor social interactions,[11-13] cognitive dysfunction,[14] increased risk of developing dementia and reduced emotional, behavioural and general social wellbeing.[15] In addition, disability in these domains is often experienced by normally-hearing significant others (SOs) living with hearing-impaired people.[16-18] Hearing impairment therefore represents an enormous burden on society and the economy.

In developed countries the most common treatment is to fit a hearing aid.[6] Despite strong evidence that hearing aid use is associated with reductions in hearing disability noted above [6 19-21] hearing aid use is remarkably low. [22 23] It is estimated that up to 30% of UK adult hearing aid owners do not use them regularly or at all.[4 24-26] International studies support these data.[8 27-29] Cost implications for the NHS are significant as they provide 80% of UK hearing aids,[25] fitting more than 300,000 new devices each year of which an estimated 20,000-120,000 are un-used. Even with global advances in technology, fitting protocols and outcome measurements,[30] there is little evidence that NHS hearing aid use and the expected gains in benefit and quality of life have improved over the last decade[19 25] and treatment continues to focus primarily on technology.

A recent systematic review of additional treatment found very low quality evidence that self-management and service delivery interventions may be of benefit in auditory rehabilitation.[31] However, the authors found no studies that examined the effect of these sorts of interventions on hearing aid use.

Reasons for hearing aid non-use are complex.[32] Research has identified psycho-social factors important for successful aural rehabilitation including; personal and societal attitudes to hearing-impairment[33] patient

For peer review only - http://bmjopen.bmj.com/site/about/guidelines.xhtml

involvement in decision making[34-37] and expectations of benefit and communication success in a range of communication situations.[38 39] Key barriers and facilitators to successful hearing aid use have been identified as being related to expectations of benefit and meaningful participation in everyday life.[40 41] The World Health Organisation's International Classification of Functioning, Disability and Health (ICF)[42] provides a functional description of difficulties (related to these expectations) experienced by hearing-impaired people and their hearing communication partners, for example, avoidance of difficult listening situations that lead to 'activity limitations' and 'participation restrictions'.

One intervention that is designed to reduce these limitations and restrictions is The Active Communication Education (ACE) programme (the focus of this paper). The ACE trains participants to develop solutions to specific difficult communication scenarios that commonly lead to their avoidance of or reduced participation in important activities. The effectiveness of ACE as an *alternative* intervention to a hearing aid has been evaluated and two small studies demonstrate ACE benefits in improving communication function and hearing related quality-of-life.[43 44] ACE effectiveness as an *adjunct* to hearing aid fitting has not been evaluated, although there is some weak evidence that supports its use in this context [44] i.e. longer-term improvements in using communication strategies being associated with hearing aid users.

A systematic review of group communication programme effectiveness conducted in 2005,[45] revealed just nine small and methodologically poor studies. The review reported weak evidence for short-term benefits related to reduced hearing disability; improvements in quality-of-life; hearing aid use and communication function[46] when interventions were delivered in concert with a hearing aid. The authors concluded that there was a clear need for large sufficiently powered randomized controlled studies to determine short and long term benefits of adult communication rehabilitation group interventions as an adjunct to hearing aid fitting. Such a study has yet to be completed. Evidence that post-dates this 2005 review does little to change the situation providing only weak additional evidence for moderate gains in hearing-loss related quality-of-life for communication-based group interventions.[47] There are indications that group

For peer review only - http://bmjopen.bmj.com/site/about/guidelines.xhtml

rehabilitation programmes like ACE have the potential to realise economic gains for service providers. For example, Abrams[48] estimated that a hearing aid plus a four-week group rehabilitation programme reduced the treatment cost per quality-adjusted life-year (QALY) gained by more than half; the cost of implementing the rehabilitation programme was less than 6% of the total rehabilitation cost per patient. Even so, with no strong evidence that such interventions delivered as an adjunct to hearing aid fitting are clinically worthwhile, they are not routinely offered in public or private hearing healthcare sectors in the UK.

In summary, there is some low quality evidence that ACE and similar programmes improve communication function and quality-of-life and these outcomes may be enhanced when delivered in conjunction with a hearing aid. Communication programmes involve a substantial commitment on the part of participants and those who run and pay for them. Recent evidence shows that it is effective and feasible to deliver ACE as an alternative intervention in Australia and Sweden.[43 44 49 50] There is now a need to establish whether reported clinical and economic benefits of ACE and communication programmes like ACE can be achieved in the context of NHS hearing aid provision. This protocol for The ACE To HEAR study (**A**ctive **C**ommunication **E**ducation **To** improve **HEAR**ing) is intended to deliver ACE to unsuccessful or struggling new NHS hearing aid users, three-months post-fitting, in order to assess whether a large RCT designed to evaluate the effectiveness of ACE in improving hearing aid benefit within the UK National Health Service (NHS) is feasible.

METHODS AND ANALYSIS

This protocol was developed and is reported according to the SPIRIT (Standard Protocol Items for Randomized Trials) statement.[51]

Study Aim:

The aim of this study is to determine the feasibility of delivering a future, full-scale randomised controlled trial (RCT) to evaluate ACE plus treatment-as-usual versus treatment-as-usual alone, within the UK National

For peer review only - http://bmjopen.bmj.com/site/about/guidelines.xhtml

Health Service (NHS) in two UK cities. Treatment-as-usual is defined as a referral from a patient's GP to audiology services to treat permanent hearing loss. It comprises up to two appointments for hearing aid fitting and a third face to face or telephone follow-up appointment.

Study objectives

Objectives will evaluate ACE delivery and trial delivery processes.

ACE Delivery Objectives

1) To assess ACE up-take rates, eligibility and acceptability of clinic location (between and within the two study sites) for participants and SOs.
2) To evaluate the level of ACE attendance and retention amongst participants randomised to the ACE arm of the study.
3) To assess acceptability of ACE with participants, SOs and audiologists.
4) To assess capability, capacity and willingness of audiology departments to support delivery of ACE within existing services.
5) To assess intervention fidelity of delivering ACE.

Trial Delivery Objectives

6) To assess RCT recruitment rates, evaluate the randomisation process and time to accrue ACE groups.
7) To assess the acceptability of study processes to participants, SOs and audiologists (i.e. those related to recruitment, the feasibility of identifying struggling hearing aid users, randomisation process, data collection, measurement of ACE fidelity and acceptability)
8) To explore patient reported outcome measures (PROMs) and estimate likely standard deviations, including quality-of-life tools (EQ-5D-5L; SF-36) and a bespoke health care resource use/acceptability/utility questionnaire for use in an intended full-scale RCT.

Study design

For peer review only

For peer review only - http://bmjopen.bmj.com/site/about/guidelines.xhtml

This study commenced on the 1st February 2017 and is of 24 months duration. The study design is a randomised controlled, open feasibility trial with embedded economic and process evaluations delivered in one audiology department in each of two UK cities. The design of the trial is shown in Figure 1.

Study setting

Study sites for this feasibility study are the Audiology Departments at York Hospital (YH), York Teaching Hospitals NHS foundation Trust, and the Bradford Royal Infirmary (BRI), Bradford Teaching Hospitals NHS Foundation Trust.

Study population

The study population will consist of adult patients aged 18 years or over, receiving treatment-as-usual delivered in one of the two participating centres. They will be considered potentially eligible if all of the following eligibility criteria apply at their 3-month post-fitting follow-up appointment:

Inclusion criteria:

a) Moderate or less than moderate benefit, defined by IOI-HA question 2.[52 53]

b) Hearing impairment: pure-tone average better ear thresholds at 500, 1000, 2000, and 4000 Hz of more than 25 dB HTL.

c) No significant self-reported history of neurological impairment.

d) Willing to provide written informed consent.

e) Able to provide written informed consent.

f) Able to take part in the intervention by understanding and using spoken English.

g) Able to self-complete the English language outcome measure tools.

h) The following inclusion criteria for Significant Others (SOs) will be assessed:

    a. A spouse or other family member who lives with or is a carer for a patient recruited to the study.

Exclusion criteria:

For peer review only - http://bmjopen.bmj.com/site/about/guidelines.xhtml

a) Severe or profound bilateral hearing impairment. Pure-tone better ear average thresholds measured at 500, 1000, 2000, and 4000 Hz of more than 85 dB HTL, since experience [43] suggests this group of patients may struggle to effectively participate in the intervention setting.

b) Significant on-going ear related health or mental health Issues that, in the audiologist's or associate audiologist's professional opinion, would preclude hearing aid fitting or attendance at ACE sessions.

c) Unable or unwilling to give written informed consent.

In addition, SOs will be excluded if they are unable or unwilling to give written informed consent.

Patients who do not have a SO or family member able to attend the ACE sessions are still eligible to participate in the study.

Sample size calculation and recruitment

As this is a feasibility study, the main purpose is to assess the acceptability and feasibility of conducting this study, with a view to designing and conducting a future full-scale trial.[54] Six ACE groups are planned with up to seven patients in each (five minimum). This leads to a maximum sample size of 44 patients and up to 44 significant others for the ACE intervention arm, and 44 in the control arm (n=88). This sample size will allow the standard deviation of the proposed outcome measures to be estimated with reasonable certainty to inform future sample size calculations.[54] Collectively, the two study sites fit approximately 4300 hearing aids a year and based on their experience we estimate 10% will require extra help at follow-up and be eligible for inclusion. Recruitment of patients commenced on the 1st April 2017 and is now underway. The recruitment window is currently planned to end on 28th February 2018 and there is potential to extend this phase until 30th April 2018 if necessary.

Randomisation

Eligible, consenting patients from the same study site who have completed baseline assessments will be randomised by a remote, centralised randomisation service (provided by York Trials Unit) in batches of 10-14 (intervention: control ratio of 1:1) using block randomisation in a single large block per batch. Following

For peer review only - http://bmjopen.bmj.com/site/about/guidelines.xhtml

randomisation, a letter outlining the next steps will be sent to participants. For those allocated to the ACE arm, this will contain an invitation for them and their SO to attend five ACE sessions, including details of the venue, times and information.

Blinding

By the nature of the interventions used within this study, blinding of the participants and facilitator is not possible. The research team responsible for data analysis and reporting will be blinded where possible.

Intervention allocation

Consenting participants will be randomly allocated to either:

a) **Treatment-as-usual:** Participants randomised to receive Treatment-as-usual will continue to receive usual care delivered by their service.

b) **ACE plus Treatment-as-usual**: Participants randomised to receive the Active Communication Education (ACE) plus Treatment-as-usual, will attend five two hour weekly sessions of the ACE programme, developed in Australia [43] and will continue to receive usual care delivered by their service.

The ACE programme will be delivered as described in the published ACE manual[55] by a trained facilitator to groups of hard of hearing people and SOs where possible. The same audiologist facilitator will deliver ACE to all groups.

ACE consists of six modules based on everyday communication activities known to be problematic for hard of hearing adults: Module 1: Communication needs analysis; Module 2: Conversation in background noise; Module 3: Conversation around the house; Module 4: Communication with difficult speakers; Module 5: Listening to other signals; and Module 6: Listening to public-address systems.

Through the use of demonstrations, practical exercises, discussion and problem solving, participants and significant others will learn individual problem-solving skills which can be applied in a range of novel

For peer review only - http://bmjopen.bmj.com/site/about/guidelines.xhtml

situations and discuss the use of communication strategies, lip reading skills, clarification skills, and assistive technology.

Intervention fidelity

Assessment of the fidelity to the ACE training of the audiologist facilitator and the delivery of the ACE programme will follow guidance from the NIH Behaviour Change Consortium, [56] and conducted in a pragmatic approach mirroring the way fidelity of an educational programme, such as ACE, would be realistically assured in an NHS context. The trainers will reflect on the training sessions; fidelity of ACE delivery will be *facilitated* by supervision of the audiologist for the first session, with feedback and self-reflection used to address any issues; fidelity of ACE delivery will then be *assessed* via a self-monitoring form recording on a 1-4 scale (poor – good) of their adherence to the ACE manual for each module across all sessions.

Intervention Compliance

Measuring compliance is challenging in complex interventions such as this, where there are a number of interacting elements (such as the influences of the ACE facilitator, participants' significant other, a varied selection of ACE module topics worked on and each individuals' perceptions of their (or their SO's) disability and treatment benefit). The intervention to be delivered is defined in the ACE manual[55] and our understanding (measurement) of what is actually delivered will be informed by the fidelity measures above, weekly attendance logs and weekly session records. ACE participants and SOs will self-report goal setting for each module and the number of goals achieved during the programme, the number of completed exercises (homework) will be reported providing an additional measure of the level of engagement or compliance with the programme that is focused on the participant and their SO.

Concurrent treatments

For peer review only - http://bmjopen.bmj.com/site/about/guidelines.xhtml

Any additional treatments identified will be available to both arms when necessary (e.g. hearing aid repairs, replacement batteries, etc.). The bespoke resource use questionnaire (see below) will allow us to monitor additional treatment accessed during the study.

Outcome Measures

Figure one shows the main processes of the trial, their relationship with the outcome measures and their timing. Data will be collected at baseline (prior to randomisation); during all ACE sessions and after ACE session 5 for ACE participants; and at an equivalent time for Treatment-as-usual participants; and approximately 3 months post-randomisation (6 months post-hearing aid fitting).

The feasibility and the potential for a future large-scale study design (the study's aim) will be measured by recording and evaluating:

ACE Delivery Outcomes:

1. Attendance: Attendance rates of participants and SOs at ACE sessions will be measured and who attends ACE session with the participant will be recorded (objective 2).
2. Fit of ACE with existing variations in service delivery models: Comparison of attendance at ACE delivered at different study sites and their satellite clinics and the effect of using telephone or face-to-face follow-up appointments will be recorded (objectives 1 and 4).
3. Can ACE be delivered as intended in the ACE protocol? This will be assessed through: (i) time taken to train the ACE facilitator successfully; (ii) number of ACE goals achieved by participants; and (iii) facilitator's adherence to the ACE protocol (fidelity) (objective 5).
4. Acceptability: Bespoke acceptability questionnaire regarding study processes, designed to explore participant and SO thoughts regarding the study including; ACE session organisation, session content, being approached and informed about the study and completing the questionnaires. The questionnaire is administered to participants and SOs at final ACE session and at a comparable time for the questions for Treatment-as-usual arm (objectives 3 and 4).

For peer review only - http://bmjopen.bmj.com/site/about/guidelines.xhtml

RCT Delivery Outcomes:

5. Recruitment: Number of follow-up cases in GP referred pathway; number of follow-up cases in GP referred pathway struggling with their hearing aid; number of and reasons for exclusions; number of patients who decline to participate and reason for declining; number who miss ACE intervention window (i.e. unable to attend an ACE group within 1-3 weeks after randomisation); number given an appointment for an ACE group session; and number of consented participants who fail to attend ACE sessions (objectives 6 and 7).
6. Allocation: Time taken to recruit and logistics of recruiting an optimally sized and located ACE group; time ACE started after randomisation (ACE intervention window); (objective 6).
7. Patient Reported Outcome Measure Data: Completion of the outcome measures below at each time-point will be recorded as well as extent of missing data within each outcome measure (objective 8)

Patient Reported Outcome Measures:

- International Outcomes Inventory for Hearing Aids (IOI-HA): [52] a seven-item questionnaire designed to evaluate the effectiveness of hearing aid treatments. The domains covered are: daily use; benefit; residual activity limitations; satisfaction; residual participation restrictions; impact on others; and quality of life. Responses are assigned a value from 1 to 5 and values summed. Higher scores indicate a more favourable outcome.
- International Outcomes Inventory for Alternative Interventions (IOI-AI): [57] a version of the IOI designed for use for non-hearing aid based interventions such as ACE, covering the same domains as the IOI-HA.
- Self-Assessment of Communication (SAC):[58] designed to measure the effect of hearing loss and hearing aid outcomes. This ten-item instrument covers questions about communication problems using a Likert

For peer review only - http://bmjopen.bmj.com/site/about/guidelines.xhtml

scale ranging from 1 ("almost never") to 5 ("practically always"). A percentage score is calculated by multiplying the raw score by 2, subtracting 20, and multiplying by 1.25.

- EQ-5D-5L:[59 60] A standardised generic instrument for describing and valuing health in terms of five dimensions (Mobility, Self-Care, Usual Activities, Pain/Discomfort, and Anxiety/Depression) using five levels of severity. Overall health on the day is also rated by the respondent on a 0–100 vertical visual analogue scale (EQ-VAS).
- Short-Form 36 (SF-36):[61] a generic health measure with 36 items assessing eight health concepts: physical functioning; role limitations due to physical problems; general health perceptions; vitality; social functioning; role limitations due to emotional problems; general mental health; and health transition

The following will be completed by participants' SOs only:

- International Outcome Inventory for Alternative Interventions: version for Significant Others (IOI-AI-SO):[56] a version of the IOI designed for use with SOs and non-hearing aid based interventions covering the same seven domains as the IOI-HA.
- International Outcomes Inventory for Hearing Aids: version for Significant Others (IOI-HA-SO):[56] an extension of the IOI-HA for use with the SO covering the same seven domains as the IOI-HA.
- Significant Other Scale for Hearing Disability (SOS-HEAR):[16] a 27-item self-report tool, which assesses third-party disability in spouses of older people with hearing impairment. It measures the effects of hearing impairment on the SO in the following domains: Changes to communication; Communication burden; Relationship changes; Going out and socializing; Emotional reactions to adaptations; Concern for partner. It uses a 5-point response scale: 0= no problem, to 4 = a complete problem.

The feasibility of collecting postal questionnaire data at each time point will be evaluated. Table 1 shows the data collection schedule.

For peer review only - http://bmjopen.bmj.com/site/about/guidelines.xhtml

Table 1: Data collection schedule: data are collected approximately three months post hearing aid fitting (Baseline); at each ACE session 1-5 for the intervention arm and at a time equivalent to ACE week 5 for the Treatment-as-usual arm; and approximately six months post hearing aid fitting. ~ ACE arm only. * Significant Others only

Study period:	Recruitment	Allocation	Post allocation					
Time point:	Baseline	0	ACE week 1	ACE week 2	ACE week 3	ACE week 4	ACE week 5	6 month
**RECRUITMENT:**								
Eligibility	•							
Informed consent	•							
Optional qualitative study consent	•							
Allocation		•						
**ASSESSMENTS:**								
Demographics	•							
IOI-HA	•						•	•
SAC	•							•
EQ-5D-5L	•							•
SF36	•							•
Resource use	•							•
IOI-AI~							•	•
ACE Participant attendance~			•	•	•	•	•	
ACE SO attendance~			•	•	•	•	•	
IOI-AI-SO~*							•	•
IOI-HA-SO*	•						•	•
SOS-HEAR*	•						•	•
Acceptability questionnaire							•	
Qualitative interviews (Participant & SO)							•	
Qualitative interviews (Audiologists)								•

For peer review only

For peer review only - http://bmjopen.bmj.com/site/about/guidelines.xhtml

Screening and enrolment

Patients attending Audiology Clinics at York Hospital (YH) and Bradford Royal Infirmary (BRI) will be approached to take part. Treatment-as-usual provided at the post hearing aid fitting follow-up will be according to site and therefore the recruitment process will vary slightly at each site:

a) York: patients will be followed up via a telephone interview. Eligibility will be checked during telephone interview and from medical records. Details of eligible and interested patients will, with their permission, be passed onto a non-ACE researcher who will post out a PIS and conduct a telephone follow-up call a few days later to see if still interested. Contact information is provided in the PIS so that the patient has opportunity to ask questions regarding the study. If willing to participate, informed consent and baseline questionnaire will be completed by post. SOs of patients who are recruited at York will receive a SO-specific PIS, consent form and baseline questionnaire by post to be returned in a freepost envelope.

b) Bradford: patients will be offered a face-to-face follow-up appointment 3-months post hearing aid fitting. Eligibility will be checked at this appointment and from medical records. Details of eligible and interested patients will be passed onto a non-ACE researcher who will provide a patient information sheet (PIS) and discuss the study. The patient will have the opportunity to ask questions and if willing, provide informed consent and complete a baseline questionnaire. If further consideration is required, the patient will be contacted by telephone call a few days later to see if still interested. In addition, if an SO attends the appointment with a patient, they will be provided with a SO-specific PIS, consent form and baseline questionnaire. Otherwise the documentation will be given to the patient to pass on to their SO or posted out.

We will monitor the proportion of patients referred to the Treatment-as-usual pathway during the study recruitment who subsequently do not attend or are not contactable by telephone for their post HA fitting

For peer review only - http://bmjopen.bmj.com/site/about/guidelines.xhtml

follow-up in order to estimate how many referrals may potentially be lost to recruitment. We will liaise with audiologists to identify reasons for non-attendance where possible.

Data collection and management

All data for the participant outcome measures will be collected by self-completed questionnaires returned by post or in secure boxes within the audiology clinics. Participants and SOs who fail to return their postal questionnaires will receive one reminder letter. Participants may also be asked to complete a questionnaire over the telephone, or asked to provide any missing data if required. Data from these paper forms will then be entered into a master database for the trial using either optical scanning techniques or entered manually.

Participants may withdraw from all or any aspects of the study without influencing their future care or treatment. A brief update of how the study is progressing will be sent out in order to maintain participant engagement with the study.

All information collected during the course of the trial will be kept strictly confidential. Information will be held securely on paper and electronically at York Trials Unit. All trial data will be identified using a unique trial identification number. Analytical datasets will not contain any identifiable information. Data will be archived for a period of at least 10 years following the end of the study.

Statistical analysis

A single analysis will be conducted at the end of the trial using Stata v13 or later. Data summaries and analyses will inform the design of a full-scale RCT of the intervention. Baseline data will be summarised by trial arm, using descriptive statistics for continuous data (mean, standard deviation (SD), median, minimum, maximum, number missing) and counts and percentages for categorical data. Recruitment rates will be reported monthly and overall, and by site. The flow of participants through the trial will be detailed in a CONSORT flow diagram and referral, consent and attendance rates will be summarised overall and by site using counts, percentages and 95% confidence intervals.

For peer review only

For peer review only - http://bmjopen.bmj.com/site/about/guidelines.xhtml

The number of ACE sessions attended will be summarised alongside any SOs who attended the sessions. Summaries will be provided overall, by site/clinic and by whether follow-up appointments were made as single or block booking. Acceptability data using Likert scales at 6 months, for participants, SOs and audiologists will be summarised separately using summary statistics and presented graphically using bar charts, by trial arm.

The number of participants withdrawing from the ACE intervention and/or the trial and any reasons for withdrawal will be summarised.

The time taken to train audiologists to deliver ACE and the number of ACE goals achieved by participants will be summarised. Fidelity scores (from self-monitoring forms) will be summarised overall and by session. The proportion of training and ACE intervention delivered as intended, as well as any adaptations to training/intervention will be reported. Variations in dose of ACE intervention will be measured through ACE attendance and attrition data.

Questionnaire return rates at each time point will be presented overall and by trial arm. PROMs at each time point will be summarised descriptively overall, by trial arm, and by ACE group for participants allocated to receive the ACE intervention. Standard deviations will be presented with 80% confidence intervals to inform future sample size calculations. The proportion of participants at the floor and ceiling of each measure, at each time point, will be reported along with the standardised response mean (SRM) to measure the sensitivity of each questionnaire to detect change. The SRM will be calculated as mean change in scores or values divided by the standard deviation in change scores.[62] Questionnaire completion times (from self-report) will be summarised as a consideration for instruments going into the full-scale evaluation. Missing data will be reported as a proportion of the total expected data set for each measure and will inform feasibility.

Qualitative data

Following delivery of all the ACE intervention sessions:

For peer review only - http://bmjopen.bmj.com/site/about/guidelines.xhtml

- The facilitator will be interviewed to explore the training and implementation process and their experiences of delivering ACE including barriers/facilitators to adhering to the ACE protocol.
- Up to three audiologists from both study sites (up to six in total) will also be interviewed, exploring the capability, capacity and willingness of their audiology departments to support the ACE study within their existing services. The acceptability of study processes will also be explored.
- A sample of 10-12 participants in the ACE intervention arm and four participants in the control arm (with their SOs if willing) will also be interviewed as soon as possible after the completion of the ACE sessions. We will purposively select participants to ensure a mix of those with good/poor hearing aid outcomes (measured at the 5th and final ACE session and equivalent control arm time point, see Fig 1. for outcome measures) as well as ensuring we include some participants who dropped out of the sessions/study, ensuring a wide range of views are collected. Control arm participants will include those who have dropped out from the study where possible, allowing us to explore reasons for this. SOs will be interviewed as part of a dyad with the participant. Semi-structured interviews will explore the acceptability of the ACE (e.g. venue, timing, content), its perceived impact (reflecting on hearing aid outcomes) with ACE intervention arm participants; and views on study processes (e.g. recruitment, outcome measures and timing) with ACE intervention and control arm participants.

Interviews will be audio-recorded, transcribed verbatim and analysed (with NVivo-11) by the research team led by CJ, using the Framework approach[63] which is particularly useful for analysing qualitative data in a pragmatic yet systematic way, where theoretical development is not needed. The steps are familiarisation, construction of a thematic framework, indexing and charting the data, mapping and interpretation.

Economic analysis and quality of life data

A full cost-effectiveness analysis will not be conducted as this is a feasibility trial thus the study is not powered to detect significant differences.

For peer review only - http://bmjopen.bmj.com/site/about/guidelines.xhtml

The costs of implementing the ACE intervention will be estimated and the potential resource implications versus usual care will be explored. Resource use will be summarised by resource use type (e.g. GP appointments, outpatient appointments) and appropriate unit costs to be applied to each resource use type will be identified. These will be sourced from a combination of local costings and national databases.[64] The costing approach will take a broad analytical perspective accounting not only for NHS costs but also for those observed by patients, though this cost will be presented separately. It is anticipated that additional resources utilised in the ACE intervention arm will largely be NHS staff time and travel/time for patients and SOs, patients and SOs use of primary and secondary NHS care, any private treatments attended, whether related to their hearing or for any other reason, changes to medication and employment or recreational activities. A draft resource use questionnaire based on these anticipated additional resources was designed for this study. The questionnaire will be developed further and tested during the feasibility trial to ensure that all relevant and necessary data can be collected to establish a reliable and valid tool with which to capture resource use for a future full economic evaluation.

Methods to estimate an incremental cost-effectiveness ratio for the ACE intervention versus Treatment-as-usual alone in terms of Quality Adjusted Life Years (QALYs) will be explored. No health-related quality of life assessment tool is currently sufficiently sensitive to all populations, and in the field of hearing health, there has been limited research to identify the most effective tool. In the UK, NICE advocates the used of the EQ-5D for generating quality adjusted life years, though it is acknowledged that this is not always the most sensitive tool for particular populations for whom the majority of its dimensions may not apply.[65] In the US analysis of a four week rehabilitation programme noted above,[48] the SF-36 was used to generate QALYs rather than the EQ-5D, with the SF-36 showing a reduction in the cost per QALY in favour of the intervention. For the present feasibility trial, both assessments will be used to elicit QALYs and a comparison will be made between the outcomes of the two measures. This will enable a decision to be made as to which tool would be most appropriate in the full-scale trial.

For peer review only - http://bmjopen.bmj.com/site/about/guidelines.xhtml

The feasibility work will also be used to help to identify any patterns of missing data and any issues relevant for sensitivity analysis which will influence statistical plans for dealing with imprecision and other uncertainties in the full RCT. For example, data can be bootstrapped to account for the expected skewness evident in economic cost data. The data collected as part of this feasibility study will be used to inform subsequent pre-trial modelling.

Adverse events

Risks within this study are considered to be minimal. It is considered highly unlikely that the ACE intervention arm will suffer any adverse consequences as a result of receiving the ACE plus Treatment-as-usual. Nevertheless, interviews with ACE participants, the ACE facilitator and ACE questionnaire data will be used to monitor this eventuality.

Trial monitoring and oversight

Due to the low risk nature of this trial, approval has been obtained to set up one independent steering and monitoring committee to undertake the roles traditionally undertaken by the Trial Steering Committee and Data Monitoring and Ethics Committee. Regular meetings of a Study Management Group will take place to oversee the progress of the study and review recruitment. We will establish a Project Advisory Panel (PAP) with between two and four hard of hearing adults or hearing spouses that will meet a minimum of five times over the duration of the project. The PAP is a group of patients, service users, carers and lay members of the public whose role is to support and advise the Study Management Group on all aspects of the study's progression and management.

Patient and Public Involvement

Three PPI activities informed the development of this application. First, a funded public engagement event about public perceptions of hearing-impairment was held at the Thackeray Medical Museum in Leeds. This event helped to inform the research question. Participants identified a need for wider availability of

For peer review only

For peer review only - http://bmjopen.bmj.com/site/about/guidelines.xhtml

treatments additional to hearing-aids and that non-technological interventions for hearing-impaired people were a priority. Delegates identified communication education as a useful addition to hearing-aid use for many hearing-impaired people and their family members. This feedback informed the study design in the following way: the choice of an interactive communication based intervention rather than an informational one; the need to ensure that routine practical information about hearing-aids and hearing-impairment is delivered consistently and checked after fitting.

Second, a focus group was held to consult with service-users on the proposed research question, study design and intervention delivery. Four participants were asked to discuss (a) study information and consent procedures, (b) factors that might encourage or discourage their participation in the proposed study such as the burden of the intervention, (c) types of communication scenarios that are important to them, (d) factors that might motivate them to be more active communicators. The outcomes informed our recruitment strategy to maximise interest, commitment and recruitment rates. The group's views helped develop study information sheets and operational components of delivering ACE.

Third, the charity Hearing Link, who have extensive experience of PPI and managing and delivering group interventions of this type, were consulted about involving public and patients in operationalising and delivering ACE. Patients and service users and carers are involved in the conduct of this study as active members of the Project Advisory Panel (PAP).

We will present the findings of this study in patient forums, Trust bulletins and PPI activities including newsletters and public interest groups who work and support older adults with hearing impairment.

Data Monitoring and management

For peer review only - http://bmjopen.bmj.com/site/about/guidelines.xhtml

Information relating to study participants will be kept confidential and managed in accordance with the Data Protection Act, NHS Caldicott Guardian, Research Governance Framework for Health and Social Care and the Research Ethics Committee (REC) approval.

Participant details will be stored on a secure password protected server located at the University of York, for the purposes of assisting in follow-ups during the study. All paper data collected from participants will be maintained in a safe secure environment at York Trials Unit. Paper records will be identified using identifiers rather than personally identifiable information. Analytical datasets will not contain any identifiable information.

The confidentiality of the participants, SOs and audiologists interviewed during their qualitative interviews will be ensured by assigning a unique identification code to electronic sound files and transcripts of interviews, known only to the qualitative researcher and appropriate members of the research team. Any quotes published will be anonymous further protecting participant confidentiality.

ETHICS AND DISSEMINATION

Ethical approval has been granted by South East Coast—Surrey Research Ethics Committee (16/LO/2012).

Since the study started in February 2017, three HRA approved amendments were added to the protocol and are included in the final version reported here:

1. Revised the fidelity check tool. This was considered a non-substantial amendment.
2. Remove inclusion criteria of less than three hours hearing aid use a day and adjusted inclusion criteria b) to include moderate benefit. We also gained approval to distribute study information flyers to study site staff and patients. These were considered substantial amendments.

For peer review only - http://bmjopen.bmj.com/site/about/guidelines.xhtml

3. Developed a study information flyer to be sent to patients not contactable by telephone for follow-up at the York study site. This was considered a non-substantial amendment.

The proposed study will be conducted in accordance with the MRC Guidelines on Good Clinical Practice in Clinical Trials.

The results from this study will be submitted to the funders, peer-reviewed journals, presented at relevant meetings/conferences and within the participating and other audiology departments. We also intend to present the findings of this study in patient forums, Trust bulletins and PPI activities including newsletters and public interest groups who work and support older adults with hearing impairment.

CONCLUSION

This will be the first RCT of this type of group communication programme in the context of a public health service and as an adjunct to hearing aid fitting. The impact of this study will ultimately be realised by a larger fully powered RCT designed to determine the effectiveness of the ACE intervention in improving hearing aid benefit for hearing aid users within the NHS GP referral for a hearing aid pathway in the UK. The outcomes of this study will inform such a RCT.

The feasibility study will be deemed successful if:

1. 70% of recruitment targets attained for all research components.
2. Study consent/retention rates and proposed sample sizes, indicate delivery of the full RCT is plausible within a 5 year study period.
3. 90% of ACE groups of 5-7 consented participants formed within the intervention window with participants attending 3 of 5 sessions.
4. Economic, acceptability, outcome measure, and fidelity evaluation data successfully collected.
5. Participants, significant-others and audiologists evaluate acceptability of the ACE and RCT positively.

(Measures with over 10% missing data maybe modified/replaced prior to the main trial)

The criteria for success will result in one of following outcomes:

1. stop: full-scale RCT not be feasible in NHS setting
2. continue: feasible with modifications

For peer review only

For peer review only - http://bmjopen.bmj.com/site/about/guidelines.xhtml

3. continue: feasible with no modifications, close monitoring
4. continue feasible as is

For peer review only

FIGURE CAPTIONS

Figure 1: ACE To HEAR Study flow diagram. The diagram was developed using CONSORT guidelines (http://www.consort-statement.org/) and indicates the main processes of the trial, their relationship with the outcome measures and their timing.

For peer review only

For peer review only - http://bmjopen.bmj.com/site/about/guidelines.xhtml

REFERENCES

1. Organisation WH. WHO global estimates on prevalence of hearing loss: Mortality and Burden of Diseases and Prevention of Blindness and Deafness. Secondary WHO global estimates on prevalence of hearing loss: Mortality and Burden of Diseases and Prevention of Blindness and Deafness. 2012. http://www.who.int/pbd/deafness/WHO_GE_HL.pdf.
2. Collins JG. Prevalence of selected chronic conditions: United States 1990-1992. Vital Health Stat 10(194) 1997.
3. Davis AC. The Prevalence of Hearing Impairmrent and Reported Hearing Disability Among Adults in Great Britain. International Journal of Epidemiology 1989;**18**(4):911-17.
4. AoHL. Hearing Matters: Taking action on hearing loss in the 21st century. http://www.actiononhearingloss.org.uk/supporting-you/policy-research-and-influencing/research/hearing-matters.aspx: Action on Hearing Loss, 2011.
5. Roth TN, Hanebuth D, Probst R. Prevalence of age-related hearing loss in Europe: a review. European Archives of Oto-Rhino-Laryngology 2011;**268**(8):1101-07.
6. Bess FH, Lichtenstein MJ, Logan SA, et al. Hearing impairment as a determinant of function in the elderly. J Am Geriatr Soc 1989;**37**(11):123-28.
7. Chia EM, Wang JJ, Rochtchina E, et al. Hearing impairment and health-related quality of life: The blue mountains hearing study. Ear and Hearing 2007;**28**:187-95.
8. Health Quality Ontario. Social isolation in community-dwelling seniors: an evidence-based analysis. Ontario health technology assessment series 2008;**8**(5):1-49.
9. Pronk, M., Deeg, D.J.H., Smits, C., et al. Prospective effects of hearing status on loneliness and depression in older persons: Identification of subgroups. International Journal of Audiology. 2011, **50**(12), pp.887-896.

For peer review only - http://bmjopen.bmj.com/site/about/guidelines.xhtml

10. Gopinath B, Schneider J, Hickson L, et al. Hearing handicap, rather than measured hearing impairment, predicts poorer quality of life over 10 years in older adults. Maturitas 2012;**72**(2):146-51.

11. Hetu R, Jones L, Getty L. The impact of acquired hearing impairment on intimate-relationships - implications for rehabilitation Audiology 1993;**32**(6):363-81.

12. Heine C, Browning C. The communication and psychosocial perceptions of older adults with sensory loss: a qualitative study. Ageing and Society 2004;**24**:113-30.

13. Heine C, Browning CJ. Communication and psychosocial consequences of sensory loss in older adults: overview and rehabilitation directions. Disability and Rehabilitation 2002;**24**(15):763-73.

14. Humes LE, Dubno JR, Gordon-Salant S, et al. Central Presbycusis: A Review and Evaluation of the Evidence. Journal of the American Academy of Audiology 2012;**23**(8):635-66.

15. Stephens D, Kramer SE. Living with hearing loss: The Process of Enablement. . London: Wiley, 2009.

16. Scarinci N, Worrall L, Hickson L. The effect of hearing impairment in older people on the spouse: Development and psychometric testing of The Significant Other Scale for Hearing Disability (SOS-HEAR). International Journal of Audiology 2009;**48**(10):671-83.

17. Wallhagen MI, Strawbridge WJ, Shema SJ, et al. Impact of self-assessed hearing loss on a spouse: A longitudinal analysis of couples. Journals of Gerontology Series B-Psychological Sciences and Social Sciences 2004;**59**(3):S190-S96.

18. Scarinci N, Worrall L, Hickson L. Factors Associated With Third-Party Disability in Spouses of Older People With Hearing Impairment. Ear and Hearing 2012;**33**(6):698-708.

19. Kochkin S. MarkeTrak VIII patients report improved quality of life with hearing aid usage. Hearing Journal 2011;**64**(6):25-32.

20. Laplante-Levesque A, Hickson L, Worrall L. Rehabilitation of Older Adults With Hearing Impairment: A Critical Review. Journal of Aging and Health 2010;**22**(2):143-53.

For peer review only - http://bmjopen.bmj.com/site/about/guidelines.xhtml

21. Ferguson MA, Kitterick PT, Chong LY, et al. Hearing aids for mild to moderate hearing loss in adults. Cochrane Database of Systematic Reviews 2017(9).

22. RNID. Royal National Institute for the Deaf. Information and resources. Secondary Royal National Institute for the Deaf. Information and resources 2011. http://www.rnid.org.uk/information_resources/

23. Davis A, Smith P, Ferguson M, et al. Acceptability, benefit and costs of early screening for hearing disability: a study of potential screening tests and models. Health Technology Assessment 2007;**11**(42).

24. Gianopoulos I, Stephens D, Davis A. Follow up of people fitted with hearing aids after adult hearing screening: the need for support after fitting. BMJ 2002;**325**.

25. Hougaard S, Ruf S. EuroTrak I: A consumer survey about hearing aids in Germany, France and the UK. First global comparative study of hearing, hearing loss, and hearing aids. The Hearing Review 2011;**18**(2):12-28.

26. Stephens D, Lewis P, Davis A, et al. Hearing aid possession in the population: Lessons from a small country. Audiology 2001;**40**(2):104-11.

27. Lupsakko TA, Kautiainen JH, Sulkava R. The non-use of hearing aids in people aged 75 years and over in the city of Kuopio in Finland. European Archives of Oto-Rhino-Laryngology 2005;**262**:165-69.

28. Popelka MM, Cruickshanks KJ, Wiley TL, et al. Low prevalence of hearing aid use among older adults with hearing loss: The epidemiology of hearing loss study. Journal of the American Geriatrics Society 1998;**46**(9):1075-78.

29. Smeeth L, Fletcher AE, Siu-Woon Ng E, et al. Reduced hearing, ownership, and use of hearing aids in elderly people in the UK-the MRC Trial of the Assessment and Management of Older People in the Community: a cross-sectional survey. The Lancet 2002;**359**(9316):1466-70.

30. Department of Health. Modernising NHS hearing aid services London: Department of Health, 2000.

31. Barker F, Mackenzie E, Elliott L, et al. Interventions to improve hearing aid use in adult auditory rehabilitation (Review). Cochrane Database of Systematic Reviews 2016; **Art. No.: CD010342**(8).

For peer review only

32. Ng JH-Y, Loke AY. Determinants of hearing aid adoption and use among the elderly: A systematic review. International Journal of Audiology 2015;**54**(5):291-300.

33. Wallhagen MI. The Stigma of Hearing Loss. Gerontologist 2010;**50**(1):66-75.

34. Laplante-Levesque A, Hickson L, Worrall L. What Makes Adults With Hearing Impairment Take Up Hearing Aids or Communication Programs and Achieve Successful Outcomes? Ear and Hearing 2012;**33**(1):79-93.

35. Laplante-Lévesque A, Hickson L, Worrall L. Factors influencing rehabilitation decisions of adults with acquired hearing impairment. International Journal of Audiology 2010;**49**(7):497-507.

36. Laplante-Levesque A, Knudsen LV, Preminger JE, et al. Hearing help-seeking and rehabilitation: Perspectives of adults with hearing impairment. International Journal of Audiology 2012;**51**(2):93-102.

37. Southall K, Gagne JP, Leroux T. Factors that influence the use of assistance technologies by older adults who have a hearing loss. International Journal of Audiology 2006;**45**(4):252-59.

38. Scarinci N, Worrall L, Hickson L. The effect of hearing impairment in older people on the spouse. International Journal of Audiology 2008;**47**(3):141-51.

39. Scarinci N, Worrall L, Hickson L. The ICF and third-party disability: Its application to spouses of older people with hearing impairment. Disability and Rehabilitation 2009;**31**(25):2088-100.

40. Gagne JP, Jennings MB, Southall K. The ICF: A Classification System and Conceptual Framework Ideal for Audiological Rehabilitation. Perspectves on Aural Rehabilitation and its Instrumentation 2009;**16**(1):8-14.

41. Lockey K, Jennings MB, Shaw L. Exploring hearing aid use in older women through narratives. International Journal of Audiology 2010;**49**(8):542-49.

42. WHO. ICF: International Classification of Fuctioning, Disability and Health. Geneva: World Health Organisation, 2001.

For peer review only - http://bmjopen.bmj.com/site/about/guidelines.xhtml

43. Hickson L, Worrall L, Scarinci N. A randomized control trial evaluating the Active Communication Education program for older people with hearing impairment. Ear and Hearing 2007;**28**(2):212-30.

44. Oberg M, Bohn T, Larsson U. Short- and Long-Term Effects of the Modified Swedish Version of the Active Communication Education (ACE) Program for Adults with Hearing Loss. Journal of the American Academy of Audiology 2014;**25**(9):848-58.

45. Hawkins DB. Effectiveness of counseling-based adult group aural rehabilitation programs: a systematic review of the evidence. Journal of the American Academy of Audiology 2005;**16**(7):485-93.

46. Chisolm TH, Abrams HB, McArdle R. Short- and long-term outcomes of adult audiological rehabilitation. Ear and Hearing 2004;**25**(5):464-77.

47. Preminger JE. Issues associated with the measurement of psychosocial benefits of group audiologic rehabilitation programs. Trends Amplif 2007;**11**(2):113-23.

48. Abrams H, Chisolm TH, McArdle R. A cost-utility analysis of adult group audiologic rehabilitation: Are the benefits worth the cost? Journal of Rehabilitation Research and Development 2002;**39**(5):549-57.

49. Hickson L, Worrall L. Beyond hearing aid fitting: improving communication for older adults. International Journal of Audiology 2003;**42**:S84-S91.

50. Oberg M, Bohn T, Larsson U, et al. A Preliminary Evaluation of the Active Communication Education Program in a Sample of 87-Year-Old Hearing Impaired Individuals. Journal of the American Academy of Audiology 2014;**25**(2):219-28.

51. Chan A, Tetzlaff JM, Altman DG, et al. Spirit 2013 statement: Defining standard protocol items for clinical trials. Annals of Internal Medicine 2013;**158**(3):200-07.

52. Cox RM, Alexander GC. The International Outcome Inventory for Hearing Aids (IOI-HA): psychometric properties of the English version. International Journal of Audiology 2002;**41**(1):30-35.

53. Cox RM, Alexander GC, Beyer CM. Norms for the international outcome inventory for hearing aids. Journal of the American Academy of Audiology 2003;**14**(8):403-13.

For peer review only - http://bmjopen.bmj.com/site/about/guidelines.xhtml

54. Teare M, Dimairo M, Shephard N, et al. Sample size requirements to estimate key design parameters from external pilot randomised controlled trials: a simulation study. Trials 2014;**15**(1):264.

55. Hickson L, Worrall L, Scarinci N, editors. *ACE: Active Communication Education.* : University of Queensland, 2015.

56. Bellg AJ, Resnick B, Minicucci DS, et al. Enhancing Treatment Fidelity in Health Behavior Change Studies: Best Practices and Recommendations From the NIH Behavior Change Consortium. Health Psychology 2004;**23**(5):441-51.

57. Noble W. Extending the IOI to significant others and to non-hearing aid-based interventions. International Journal of Audiology 2002;**41**(1):27-29.

58. Schow RL, Nerbonne MA. communication screening profile - use with elderly clients. Ear and Hearing 1982;**3**(3):135-47.

59. Herdman M, Gudex C, Lloyd A, et al. Development and preliminary testing of the new five-level version of EQ-5D (EQ-5D-5L). Quality of Life Research. Quality of Life Research 2011;**20**(10):1727-36.

60. Janssen M, Pickard A, Golicki D, et al. Measurement properties of the EQ-5D-5L compared to the EQ-5D-3L across eight patient groups: a multi-country study. Quality of Life Research 2013;**22**(7):1717-27.

61. Ware J, Sherbourne C. The MOS 36-item short-form health survey (SF-36) 1: conceptual framework and item selection. Med Care 1992;**30**:473-83.

62. Ritchie J, Lewis J. *Qualitative Research Practice*. London: Sage Publications, 2003.

63. Stratford PW, Binkley J, Solomon P, et al. Defining the minimum level of detectable change for the Roland-Morris questionnaire. Physical Therapy 1996;**76**(4):359-65.

64. Curtis L. Unit Costs of Health and Social Care 2014, 2014.

65. NICE. Guide to the Methods of Technology Appraisal.: National Institute of Health and Clinical Excellence: London, 2013.

For peer review only

For peer review only - http://bmjopen.bmj.com/site/about/guidelines.xhtml

Contributorship Statement

NJT led on the conception, design and writing of the study and study protocol with substantial contributions to the design, writing, critical review of intellectual content and final manuscript approval from JW, CJ, KC, AF, LH, LC, KB, CF, CM,RG, KI and LG. All authors agree to be accountable for their work. As Principle Investigator, NJT takes overall responsibility for the work.

KC provided statistical expertise in the study design and development stages of the project and the protocol. CF provided further essential statistical advice and expertise on the study protocol.

JW, CJ and LC made substantial contributions to the trial design and management. AF was involved in all aspects of the study and LG provided particular input to PPI. KB was specifically responsible for the health economic aspects of the study design and KI and RG were responsible for aspects specific to Trust's service delivery, providing expert clinical support.

Data Sharing Statement

There will be no additional unpublished data available for this study.

Funding Statement

This paper presents independent research funded by the National Institute for Health Research (NIHR) under its Research for Patient Benefit (RfPB) Programme (Grant Reference Number PB-PG-0215-36147). The views expressed are those of the author(s) and not necessarily those of the NHS, the NIHR or the Department of Health and Social Care..

Competing Interest Statement

None Declared

For peer review only - http://bmjopen.bmj.com/site/about/guidelines.xhtml

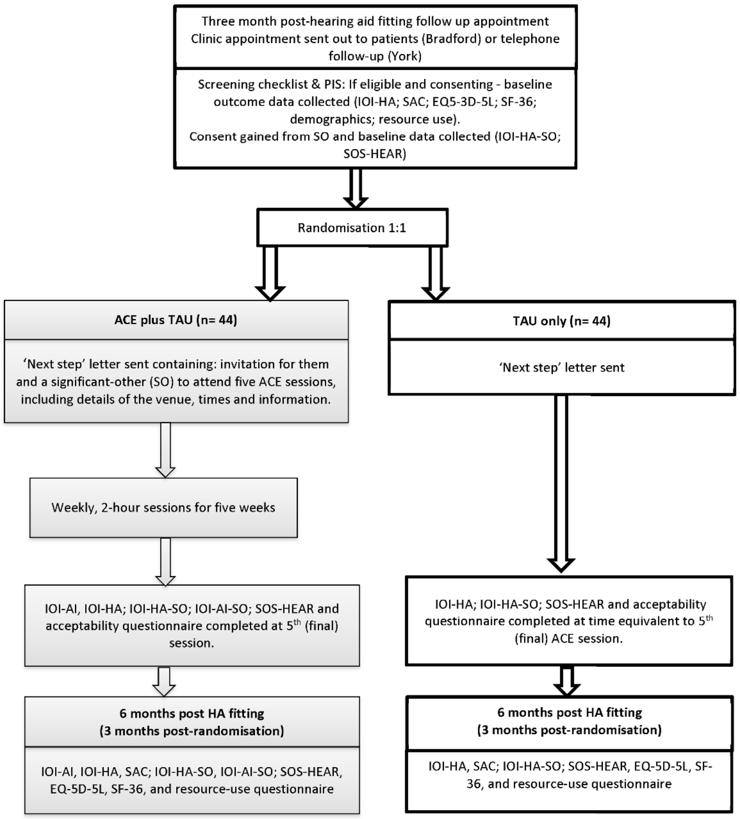

209x297mm (300 x 300 DPI)

STANDARD PROTOCOL ITEMS: RECOMMENDATIONS FOR INTERVENTIONAL TRIALS

SPIRIT 2013 Checklist: Recommended items to address in a clinical trial protocol and related documents*

Section/item	Item No	Description
**Administrative information**		
Title	1	Descriptive title identifying the study design, population, interventions, and, if applicable, trial acronym [P1, L1-3]
Trial registration	2a	Trial identifier and registry name. If not yet registered, name of intended registry [P2, L23]
	2b	All items from the World Health Organization Trial Registration Data Set. (All relevant WHO items for the current stage of the study are contained in the ISRCTN registry: URL is given on [P2, L23])
Protocol version	3	Date and version identifier [P1, L24]
Funding	4	Sources and types of financial, material, and other support [P33, L14]
Roles and responsibilities	5a	Names, affiliations, and roles of protocol contributors [P1 and P33, L1]
	5b	Name and contact information for the trial sponsor [P1, L23]
	5c	Role of study sponsor and funders, if any, in study design; collection, management, analysis, and interpretation of data; writing of the report; and the decision to submit the report for publication, including whether they will have ultimate authority over any of these activities [None]
	5d	Composition, roles, and responsibilities of the coordinating centre, steering committee, endpoint adjudication committee, data management team, and other individuals or groups overseeing the trial, if applicable (see Item 21a for data monitoring committee) [P21, L11]
**Introduction**		
Background and rationale	6a	Description of research question and justification for undertaking the trial, including summary of relevant studies (published and unpublished) examining benefits and harms for each intervention [P4, L1-P6, L18]]
	6b	Explanation for choice of comparators [P6, L14 - P7,L3]
Objectives	7	Specific objectives or hypotheses [P7, L4-22]

For peer review only

Trial design	8	Description of trial design including type of trial (eg, parallel group, crossover, factorial, single group), allocation ratio, and framework (eg, superiority, equivalence, noninferiority, exploratory) [P8, L1-3]

Methods: Participants, interventions, and outcomes

Study setting	9	Description of study settings (eg, community clinic, academic hospital) and list of countries where data will be collected. Reference to where list of study sites can be obtained [P8, L4-7]
Eligibility criteria	10	Inclusion and exclusion criteria for participants. If applicable, eligibility criteria for study centres and individuals who will perform the interventions (eg, surgeons, psychotherapists) [P8, L12-23, P9, L1-9]
Interventions	11a	Interventions for each group with sufficient detail to allow replication, including how and when they will be administered [P10, L7-23, P11, L1-2]
	11b	Criteria for discontinuing or modifying allocated interventions for a given trial participant (eg, drug dose change in response to harms, participant request, or improving/worsening disease) N/A
	11c	Strategies to improve adherence to intervention protocols, and any procedures for monitoring adherence (eg, drug tablet return, laboratory tests) N/A
	11d	Relevant concomitant care and interventions that are permitted or prohibited during the trial [P11, L22, P12, L1-3]
Outcomes	12	Primary, secondary, and other outcomes, including the specific measurement variable (eg, systolic blood pressure), analysis metric (eg, change from baseline, final value, time to event), method of aggregation (eg, median, proportion), and time point for each outcome. Explanation of the clinical relevance of chosen efficacy and harm outcomes is strongly recommended [P12, L4-24, , P13, L1-23, P14, L1-23]
Participant timeline	13	Time schedule of enrolment, interventions (including any run-ins and washouts), assessments, and visits for participants. A schematic diagram is highly recommended (see Figure 1) [P26]
Sample size	14	Estimated number of participants needed to achieve study objectives and how it was determined, including clinical and statistical assumptions supporting any sample size calculations [P9, L10-20]
Recruitment	15	Strategies for achieving adequate participant enrolment to reach target sample size [P9, L10-20]

Methods: Assignment of interventions (for controlled trials)

Allocation:

Sequence generation	16a	Method of generating the allocation sequence (eg, computer-generated random numbers), and list of any factors for stratification. To reduce predictability of a random sequence, details of any planned restriction (eg, blocking) should be provided in a separate document that is unavailable to those who enrol participants or assign interventions ??? [P9, L21-24, P10, L1-3]
Allocation concealment mechanism	16b	Mechanism of implementing the allocation sequence (eg, central telephone; sequentially numbered, opaque, sealed envelopes), describing any steps to conceal the sequence until interventions are assigned [P9, L22-23]
Implementation	16c	Who will generate the allocation sequence, who will enrol participants, and who will assign participants to interventions [P9, L22-23]
Blinding (masking)	17a	Who will be blinded after assignment to interventions (eg, trial participants, care providers, outcome assessors, data analysts), and how [P10, L4-6]
	17b	If blinded, circumstances under which unblinding is permissible, and procedure for revealing a participant's allocated intervention during the trial N/A

Methods: Data collection, management, and analysis

Data collection methods	18a	Plans for assessment and collection of outcome, baseline, and other trial data, including any related processes to promote data quality (eg, duplicate measurements, training of assessors) and a description of study instruments (eg, questionnaires, laboratory tests) along with their reliability and validity, if known. Reference to where data collection forms can be found, if not in the protocol [P16, L3-21]
	18b	Plans to promote participant retention and complete follow-up, including list of any outcome data to be collected for participants who discontinue or deviate from intervention protocols [P17, L5-11]
Data management	19	Plans for data entry, coding, security, and storage, including any related processes to promote data quality (eg, double data entry; range checks for data values). Reference to where details of data management procedures can be found, if not in the protocol [P17, L13-16]
Statistical methods	20a	Statistical methods for analysing primary and secondary outcomes. Reference to where other details of the statistical analysis plan can be found, if not in the protocol [P17, L16-24, P18, L1-22]
	20b	Methods for any additional analyses (eg, subgroup and adjusted analyses) N/A
	20c	Definition of analysis population relating to protocol non-adherence (eg, as randomised analysis), and any statistical methods to handle missing data (eg, multiple imputation) [P18, L21-22]

Methods: Monitoring

Data monitoring	21a	Composition of data monitoring committee (DMC); summary of its role and reporting structure; statement of whether it is independent from the sponsor and competing interests; and reference to where further details about its charter can be found, if not in the protocol. Alternatively, an explanation of why a DMC is not needed [P21, L11-19]
	21b	Description of any interim analyses and stopping guidelines, including who will have access to these interim results and make the final decision to terminate the trial [N/A]
Harms	22	Plans for collecting, assessing, reporting, and managing solicited and spontaneously reported adverse events and other unintended effects of trial interventions or trial conduct [P20, L6-10]
Auditing	23	Frequency and procedures for auditing trial conduct, if any, and whether the process will be independent from investigators and the sponsor [P21, L12-19]

Ethics and dissemination

Research ethics approval	24	Plans for seeking research ethics committee/institutional review board (REC/IRB) approval [P23, L14-15]
Protocol amendments	25	Plans for communicating important protocol modifications (eg, changes to eligibility criteria, outcomes, analyses) to relevant parties (eg, investigators, REC/IRBs, trial participants, trial registries, journals, regulators) [P23, L15-22, P24, L1-2]
Consent or assent	26a	Who will obtain informed consent or assent from potential trial participants or authorised surrogates, and how (see Item 32) [P16, L5-12, P16, L13-21]
	26b	Additional consent provisions for collection and use of participant data and biological specimens in ancillary studies, if applicable N/A
Confidentiality	27	How personal information about potential and enrolled participants will be collected, shared, and maintained in order to protect confidentiality before, during, and after the trial [P117, L13-16]
Declaration of interests	28	Financial and other competing interests for principal investigators for the overall trial and each study site [P33, L19-20]
Access to data	29	Statement of who will have access to the final trial dataset, and disclosure of contractual agreements that limit such access for investigators [P17, L13-16 and P22, L21 – P23, L1-12]]
Ancillary and post-trial care	30	Provisions, if any, for ancillary and post-trial care, and for compensation to those who suffer harm from trial participation N/A

For peer review only

Dissemination policy	31a	Plans for investigators and sponsor to communicate trial results to participants, healthcare professionals, the public, and other relevant groups (eg, via publication, reporting in results databases, or other data sharing arrangements), including any publication restrictions [P22, L18-19, P24, L5-8]
	31b	Authorship eligibility guidelines and any intended use of professional writers N/A
	31c	Plans, if any, for granting public access to the full protocol, participant-level dataset, and statistical code N/A
**Appendices**		
Informed consent materials	32	Model consent form and other related documentation given to participants and authorised surrogates
Biological specimens	33	Plans for collection, laboratory evaluation, and storage of biological specimens for genetic or molecular analysis in the current trial and for future use in ancillary studies, if applicable N/A

*It is strongly recommended that this checklist be read in conjunction with the SPIRIT 2013 Explanation & Elaboration for important clarification on the items. Amendments to the protocol should be tracked and dated. The SPIRIT checklist is copyrighted by the SPIRIT Group under the Creative Commons "Attribution-NonCommercial-NoDerivs 3.0 Unported" license.

For peer review only
